# Changes over time in latent patterns of childhood-to-adulthood BMI development in Great Britain: evidence from three cohorts born in 1946, 1958, and 1970

**DOI:** 10.1186/s12916-021-01969-8

**Published:** 2021-04-21

**Authors:** T. Norris, M. Hamer, R. Hardy, L. Li, K. K. Ong, G. B. Ploubidis, R. Viner, W. Johnson

**Affiliations:** 1 School of Sport Exercise and Health Sciences, Loughborough University, Loughborough, UK; 2 UCL Institute Sport Exercise Health , Division Surgery Interventional Science, London, UK; 3 UCL Institute of Education, London, UK; 4 UCL Great Ormond Street Institute of Child Health, London, UK; 5 MRC Epidemiology Unit, University of Cambridge, Cambridge, UK; 6 Centre for Longitudinal Studies, Department of Social Science, University College London, London, UK

**Keywords:** Body mass index, Secular, Cohort, Obesity, Rapid, Trajectory

## Abstract

**Background:**

Most studies on secular trends in body mass index (BMI) are cross-sectional and the few longitudinal studies have typically only investigated changes over time in mean BMI trajectories. We aimed to describe how the evolution of the obesity epidemic in Great Britain reflects shifts in the proportion of the population demonstrating different latent patterns of childhood-to-adulthood BMI development.

**Methods:**

We used pooled serial BMI data from 25,655 participants in three British cohorts: the 1946 National Survey of Health and Development (NSHD), 1958 National Child Development Study (NCDS), and 1970 British Cohort Study (BCS). Sex-specific growth mixture models captured latent patterns of BMI development between 11 and 42 years. The classes were characterised in terms of their birth cohort composition.

**Results:**

The best models had four classes, broadly similar for both sexes. The ‘lowest’ class (57% of males; 47% of females) represents the normal weight sub-population, the ‘middle’ class (16%; 15%) represents the sub-population who likely develop overweight in early/mid-adulthood, and the ‘highest’ class (6%; 9%) represents those who likely develop obesity in early/mid-adulthood. The remaining class (21%; 29%) reflects a sub-population with rapidly ‘increasing’ BMI between 11 and 42 years. Both sexes in the 1958 NCDS had greater odds of being in the ‘highest’ class compared to their peers in the 1946 NSHD but did not have greater odds of being in the ‘increasing’ class. Conversely, males and females in the 1970 BCS had 2.78 (2.15, 3.60) and 1.87 (1.53, 2.28), respectively, times higher odds of being in the ‘increasing’ class.

**Conclusions:**

Our results suggest that the obesity epidemic in Great Britain reflects not only an upward shift in BMI trajectories but also a more recent increase in the number of individuals demonstrating more rapid weight gain, from normal weight to overweight, across the second, third, and fourth decades of life.

**Supplementary Information:**

The online version contains supplementary material available at 10.1186/s12916-021-01969-8.

## Background

According to the latest data from the Non-Communicable Diseases Risk Factor Collaboration, the number of adults worldwide with obesity increased from approximately 100 million to 671 million between 1975 and 2016, with an additional 1.3 billion in the overweight range [1]. Whilst repeated cross-sectional surveys can be used to estimate changes over time in the prevalence of obesity, longitudinal data from multiple cohorts born at different points in time are needed to document secular trends in the age-related process of obesity development. Our 2015 paper on this topic used serial body mass index (BMI) data from the series of nationally representative birth cohort studies in the United Kingdom (UK), born between 1946 and 2001 [2]. We observed a positive skewing of the BMI distribution at increasingly younger ages in more recently born cohorts, as well as a progressively younger age of entry into the overweight range. Like nearly all the obesity secular trend literature, this paper did not, however, consider how the evolution of the obesity epidemic might reflect shifts in the proportion of the population demonstrating different (and perhaps new) patterns of childhood to adulthood BMI development.

Rather than the obesity epidemic reflecting the skewing of BMI [3–6], it might be that the distribution is bi-modal, reflecting two sub-populations. This hypothesis is compatible with the classic heuristic model of Ravussin and Bouchard [7], which shows how the effect of high genetic susceptibility to obesity is only unmasked in environments that are highly obesogenic. In this scenario, a larger sub-population might be ‘obesity-resilient’, and a smaller sub-population with higher BMI values might be ‘obesity-prone’. In their 2014 paper, Sperrin et al. [8] developed a growth mixture model to identify latent BMI classes or sub-populations from pseudo-panel data derived from annual cross-sectional surveys of the Health Survey for England. They identified a normal BMI ‘resistant’ sub-population and a high BMI ‘fatter’ sub-population; approximately 24% of men and 34 of women were in the ‘fatter’ sub-population. Between 1992 and 2010, mean BMI in the ‘resistant’ group remained relatively stable, but mean BMI in the ‘fatter’ group increased rapidly by approximately 3.5 kg/m^2^. This paper provided the first evidence on how secular trends in BMI might be explained by the existence of sub-populations but was limited by using panel data that does not represent a true longitudinal cohort. This meant that the authors could not identify latent classes of individuals who share similar patterns of BMI development over age, and then document how birth year was related to the probability of being in each class.

A recent paper by Nedelec et al. used pooled BMI data between 2 and 20 years of age from the 1966 and 1986 Northern Finland Birth Cohorts and identified four latent classes or sub-populations of individuals [9]. The average BMI trajectories for each class were roughly parallel to each other for most of the age range, effectively splitting the BMI distribution into just below average weight, average weight, just above average weight, and obese. Interestingly, the proportions of the 1966 and 1986 birth cohorts in the obesity class were similar (3.4 and 4.0%) suggesting only a small temporal shift in the number of children with this type of deleterious BMI trajectory. In addition to the obesity epidemic reflecting an upward shift in BMI trajectories, we would also expect there to be an increase in the number of children with rapid BMI gain [10], from normal to high values. Epidemiologically these are perhaps the most important group as they have worse cardiometabolic disease outcomes than children whose BMI is stable and high [11]. The Nedelec et al. study may not have found such a class because of how they specified their latent class trajectory model (e.g. whether variances were allowed to differ across time and classes) or because data beyond 20 years are needed to capture the sub-population of individuals with rapid childhood to adulthood BMI gain.

Using pooled data from three nationally representative cohorts born in 1946, 1958, and 1970, we aimed to describe how the evolution of the obesity epidemic in Great Britain reflects shifts in the proportion of the population demonstrating different latent patterns of BMI development between 11 and 42 years of age.

## Methods

### Samples

The three British birth cohort studies used in these analyses have been previously described in detail elsewhere [12–14] and were designed to be nationally representative when initiated. The MRC National Survey of Health and Development (NSHD) was initiated in 1946 and recruited 5362 participants. The National Child Development Study (NCDS) was initiated in 1958 and recruited 17,416 participants. The 1970 British Cohort Study (BCS70) was initiated in 1970 and recruited 16,571 participants. All of the studies have received ethical approval and obtained informed parental and/or participant consent, both of which cover the secondary analyses reported here; this information is available from the study websites and/or cohort profiles [12–15].

### Serial BMI data

As described elsewhere [2], serial BMI (kg/m^2^) was derived in each study from measured or self-reported weight and height collected at the target ages 11, 15, 26 (self-report), 36, and 43 years in the 1946 NSHD; 11, 16, 23 (self-report), 33, and 42 (self-report) years in the 1958 NCDS; and 10, 16 (one-third self-report), 26 (self-report), 34 (self-report), and 42 (self-report) years in the 1970 BCS. The distribution of ages at each measurement sweep can be seen in Additional file 1: Tables S1 and S2.

There were 15,888 observations of BMI from 3693 participants in the 1946 NSHD, with 85% of the sample having four or more observations. There were 51,540 observations of BMI from 12,519 participants in the 1958 NCDS, with 81% of the sample having four or more observations. Finally, there were 36,501 observations of BMI from 9443 participants in the 1970 BCS, with 71% of the sample having four or more observations. In total, there were 103,929 observations of BMI from 25,655 participants.

Table S3 shows the differences in several selected characteristics between the sample included versus those excluded for having less than 3 BMI measurements.

### Statistical analysis

#### Patterns of BMI development

Unconditional growth mixture models [16] were developed to identify distinct groups of individuals who had similar BMI trajectories between 11 and 42 years of age. Due to the observed differences in life course BMI trajectories between sexes, an a priori decision was made to develop sex-specific growth mixture models, as has been done elsewhere [17, 18]. We developed our mixture model specification in a series of steps, with the aim to improve the Bayesian Information Criterion (BIC) and ignoring the entropy statistic, as this is not a measure of model fit [19, 20] whilst also retaining theoretical plausibility. The age scale was centred at visit 3 (23.64 years in males and 26.00 years in females) to aid numerical stability. Model development considered several age functions for the trajectory shape, including linear, freed-loading, quadratic, and cubic polynomials. For the cubic polynomial models to converge, the variance of the quadratic and cubic term had to be constrained to be zero (i.e. no-between child differences in quadratic or cubic growth). Nonetheless, as shown in Tables S4 and S5, the cubic polynomial function provided the best fit for the data, particularly for the 3–7 class solutions. Using these cubic polynomial models, we then tried relaxing some of the main default constraints implemented by Mplus.

Firstly, heteroskedasticity in BMI residual variances was assumed across sweeps. We then tested models that allowed the residual variances/errors to differ across the classes, in line with previous research [21]. We then attempted to extend the models to include a within-class autocorrelation structure for the residual variances/errors, as recommended by Gilthorpe et al. [22]. Finally, we tested models that allowed the variance of the latent intercept of the growth curve function to differ between classes, in line with previous research [2, 3]. In summary, the best fitting model included a cubic polynomial function of age, within-class heteroscedastic errors, and a first-order autoregressive structure (AR1) to model autocorrelation. We tested models with the number of classes ranging from 1 to 7 classes and the best class solution was selected based firstly on model fit (i.e. Bayesian Information Criterion (BIC)), quality of classification or separation between the classes (e.g. entropy), and plausibility and interpretability of the average trajectories (see Additional file 1: Tables S4-S7). A plot showing the average trajectories superimposed on the International Obesity Task Force (IOTF) ranges for obesity, overweight, normal weight, and thinness [23] was produced.

Further details about the implementation of the growth mixture model can be found in Additional file 1: Text S1.

#### Cohort differences in class membership

We employed the ‘3-step’ approach developed by Vermunt [24], using the R3STEP option in Mplus. This enables relationships between the latent class variable and a set of observed predictor variables to be examined whilst appropriately incorporating the classification error which occurs during the mixture modelling process. Steps 1 and 2 refer to the process outlined above in which the growth mixture model is estimated (step 1) and most likely class membership is assigned using the latent class posterior distribution obtained during the first step (step 2). In step 3, the most likely class is regressed (using multinomial regression) on predictor variables, in this instance ‘cohort’, taking into account the misclassification observed in the second step. Mplus then provides odds ratios (ORs) and 95% confidence intervals for class membership according to cohort status, with 1946 NSHD cohort as the reference (see Additional file 2: Text S1 and S2 for input code and output).

As a sensitivity analyses, we tested the robustness of the association between cohort and class membership when a 5-trajectory-class solution was assumed.

Analyses were performed in Stata version 15 (Stata Corp, College Station, TX) and Mplus version 8.3.

## Results

Descriptive characteristics of the sample are presented in Table 1, with Additional file 1: Tables S8 and S9 displaying descriptive characteristics for males and females, respectively, stratified by BMI trajectory class. Table S3 also shows a comparison of the included sample vs those excluded for having < 3 BMI measurements. The included sample was more likely to be White British and from more educated parents with a higher social class.

**Table 1:** Description of the study sample (*n* = 25 655)

		*Total sample* *(n = 25 655)*		*NSHD 1946* *(n = 3 693)*		*1958 NCDS* *(n = 12 519)*		*1970 BCS* *(n = 9 443)*	
			Missing (*n* (%))		Missing (*n* (%))		Missing (*n* (%))		Missing (*n* (%))
**Sex**									
*Males*	*n* (%)	12 465 (48.6)	–	1 872 (50.7)	–	6 291 (50.3)	–	4 302 (45.6)	–
*Females*	*n* (%)	13 190 (51.4)		1 821 (49.3)		6 228 (49.7)		5 141 (54.4)	
**Ethnicity**									
*White British*	*n* (%)	24 884 (97.4)	103 (0.4)	3 693 (100)	0	12 235 (97.8)	6 (0.05)	8 956 (95.8)	97 (1.03)
Other^a^	*n* (%)	668 (2.6)		0		278 (2.2)		390 (4.2)	
**Birthweight (kg)**	Mean (SD)	3.3 (0.5)	1 686 (6.6)	3.4 (0.5)	14 (0.04)	3.3 (0.5)	1 013 (8.1)	3.3 (0.5)	659 (7.0)
**Childhood social class**									
*Professional*	*n* (%)	1 190 (5.3)	3 031 (11.8)	205 (6.3)	447 (12.1)	505 (4.7)	1 683 (13.4)	480 (5.6)	901 (9.5)
*Intermediate*	*n* (%)	5 032 (22.2)		637 (19.6)		2 191 (20.2)		2204 (25.8)	
*Skilled non-manual*	*n* (%)	2 630 (11.6)		513 (15.8)		1 163 (10.7)		954 (11.2)	
*Skilled manual*	*n* (%)	8 901 (39.3)		1 088 (33.5)		4 475 (41.3)		3 338 (39.1)	
*Partly skilled manual*	*n* (%)	3 301 (14.6)		618 (19.0)		1 600 (14.8)		1 083 (12.7)	
*Unskilled manual*	*n* (%)	1 570 (6.9)		185 (5.7)		902 (8.3)		483 (5.7)	
**Mother left education at mandatory leaving age**									
*Yes*	*n* (%)	16 862 (69.5)	1388 (5.4)	2 418 (71.3)	301 (8.2)	8 792 (74.0)	637 (5.1)	5652 (62.9)	450 (4.8)
**Father left education at mandatory leaving age**									
*Yes*	*n* (%)	15 744 (69.4)	2 955 (11.5)	2 313 (69.0)	342 (9.3)	3 029 (74.5)	635 (5.1)	2 889 (61.3)	1 978 (21.0)

^a^Other ethnicities: White other, Mixed race, Indian, Pakistani, Bangladeshi, Other Asian, Caribbean, African, Other Black, Chinese

### Latent BMI trajectory classes

**Fig. 1:**
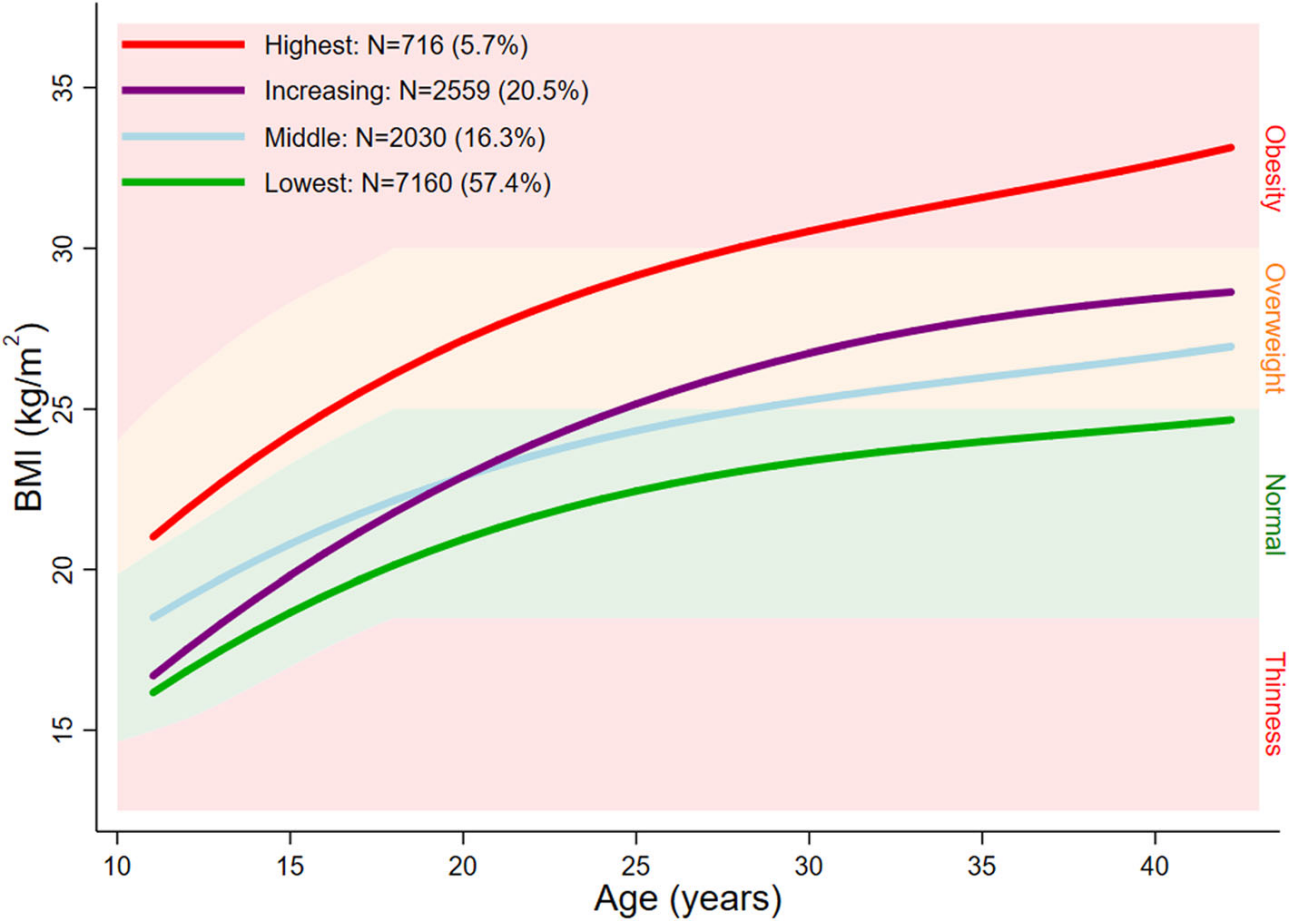
Average fitted trajectories from the final mixture model superimposed on thinness, normal weight, overweight, and obesity ranges: males

**Fig. 2:**
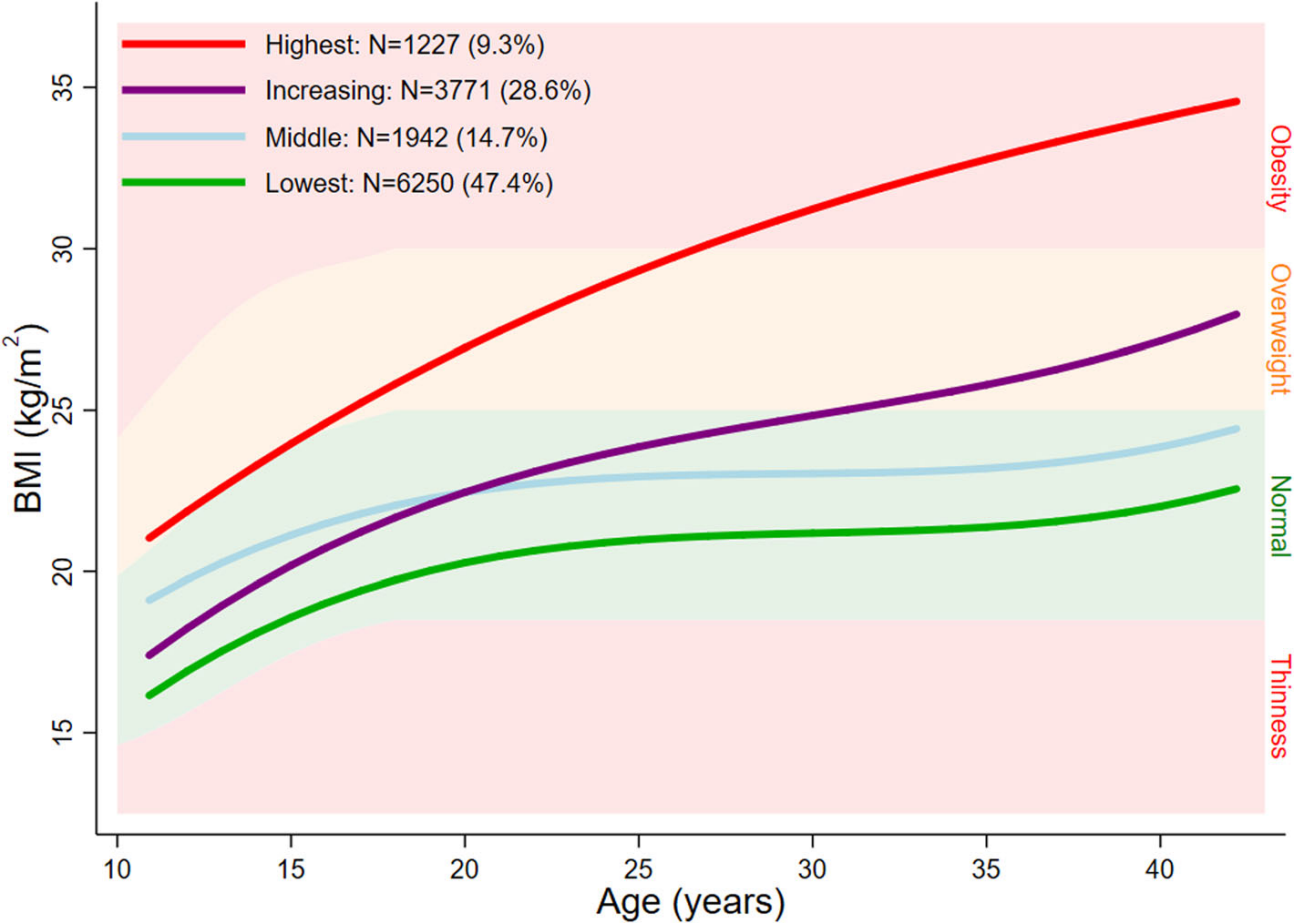
Average fitted trajectories from the final mixture model superimposed on thinness, normal weight, overweight, and obesity ranges: females

A mixture model with 4 classes provided the best representation of the serial BMI data and the most plausible solution, for males and females respectively. Figures 1 and 2 show the average fitted trajectories against the IOTF weight status ranges. The entropy statistic for the final model was 0.51 and 0.57 for males and females, respectively. The Mplus input file and relevant extracts from the mixture modelling output is included in Additional file 2: Text 1 and Text 2 and plots of posterior probabilities for each of the classes are included in Additional file 1: Figure S1 and S2.

#### Class 1: ‘Lowest’

This class comprised 57.4% and 47.4% of males and females, respectively. In both sexes, the average trajectory did not deviate from the normal weight range between 11 and 42 years. This trajectory class has been labelled ‘Lowest’.

#### Class 2: ‘Middle’

In males, this class was characterised by an average trajectory which increased from a higher normal weight (compared to class 1) at age 11 years to mid-overweight at 42 years. In females, the average trajectory maintained a position at the higher end of the normal weight range. This trajectory class has been labelled ‘Middle’ and comprised 16.3% of all males and 14.7% of all females.

#### Class 3: ‘Increasing’

The average trajectory of this class, in both sexes, was characterised by an increase from the lower half of the normal weight range to the upper half of the overweight range. This trajectory class has been labelled ‘Increasing’ and comprised 20.6% of all males 28.6% of all females.

#### Class 4: ‘Highest’

Class 4 comprised 5.8% of males and 9.3% of females. For males and females, this class was characterised by a trajectory which increased from mild overweight at 11 years to obesity at 42 years, with greater increases observed in females. Accordingly, this trajectory class has been labelled ‘Highest’.

### Cohort differences in class membership

Proportions of each cohort assigned to each class and odds ratios for class membership are reported in Table 2. In both sexes, the largest proportion of each cohort was assigned to the ‘Lowest’ class. For example, 63.5%, 59.7%, and 51.4% of males in the 1946 NSHD, 1958 NCDS, and 1970 BCS, respectively, were in this class. Similar proportions of each cohort were in the ‘Middle’ class, with 15.8%, 17.4%, and 15.0% of males and 15.1%, 16.3%, and 12.8% of females in the NSHD, NCDS, and BCS, respectively in this class. This resulted in small ORs (with 95% CIs straddling the null) for the odds of being in the ‘Middle’ trajectory (versus ‘Lowest) in both the 1958 NCDS and 1970 BCS (relative to 1946 NSHD).

**Table 2:** Proportion of each cohort assigned to each class and odds ratios for class membership

BMI trajectory class membership	*Class 1: Lowest*		*Class 2: Middle*		*Class 3: Increasing*		*Class 4: Highest*	
	*N* (%)	OR (95% CI)	*N* (%)	OR (95% CI)	*N* (%)	OR (95% CI)	*N* (%)	OR (95% CI)
Males (*n* = 12 465)								
*NSHD*	1 188 (63.5)	(ref)	296 (15.8)	(ref)	328 (17.5)	(ref)	60 (3.2)	(ref)
*NCDS*	3 757 (59.7)	–	1 093 (17.4)	1.22 (0.96, 1.54)	1 083 (17.2)	0.99 (0.76, 1.29)	358 (5.7)	2.23 (1.53, 3.25)
*BCS*	2 209 (51.3)	–	643 (15.0)	1.16 (0.88, 1.52)	1 150 (26.7)	2.78 (2.15, 3.60)	300 (7.0)	3.67 (2.50, 5.39)
Females (*n* = 13,190)								
*NSHD*	966 (53.0)	(ref)	274 (15.1)	(ref)	460 (25.3)	(ref)	121 (6.6)	(ref)
*NCDS*	3 036 (48.7)	–	1 012 (16.2)	1.26 (0.99, 1.60)	1 629 (26.2)	1.15 (0.94, 1.40)	551 (8.9)	1.58 (1.22, 2.03)
*BCS*	2 248 (43.7)	–	656 (12.8)	0.93 (0.71, 1.22)	1 682 (32.7)	1.87 (1.53, 2.28)	555 (10.8)	2.20 (1.71, 2.85)

*OR odds ratios for class membership, using ‘lowest’ class as referent class and NSHD as the referent cohort

A very similar proportion of the 1958 NCDS were assigned to the ‘Increasing’ class compared to the 1946 NSHD. For example, 17.2% and 26.2% of males and females, respectively, in the 1958 NCDS were in this class, compared to 17.5% and 25.3% of males and females in the 1946 NSHD. As a result, ORs for being in this class (compared to the ‘Lowest’) in the 1958 NCDS were small and 95% CIs straddled the null. The 1958 NCDS did, however, have a higher proportion of both sexes classified in the ‘Highest’ class compared to the 1946 NSHD, resulting in a OR of 2.23 (95% CI 1.53, 3.25) and 1.58 (95% CI 1.22, 2.03) in males and females, respectively. In the 1970 BCS, however, a greater proportion of males (26.7%) and females (32.7%) were assigned to the ‘Increasing’ trajectory class. For males, this resulted in an OR of 2.78 (95% CI 2.15, 3.60) of being assigned to the ‘Increasing’ class (versus ‘Lowest’) compared to the 1946 NSHD. For females the OR was 1.87 (95% CI 1.53, 2.28). Finally, the odds of being assigned to the ‘Highest’ trajectory class (versus ‘Lowest’) were over 3.5 times higher for males in the 1970 BCS, relative to the 1946 NSHD (OR 3.67; 95% CI 2.50, 5.39) and more than twice as higher in females in the BCS (OR 2.20; 95% CI 1.71, 2.85).

We tested the robustness of these findings by repeating the analysis using the 5-class solution and similar findings were observed (see: Additional file 1: Tables S10 and S11).

## Discussion

This study utilised longitudinal BMI data from three British birth cohort studies to identify distinct groups of individuals who share similar child-to-adulthood BMI trajectories. In both sexes, we observed the expected low and middle trajectories as well as another much higher trajectory class. In addition, we observed a trajectory class (comprising 20% of males and almost 30% of females) which was characterised by a rapid increase in BMI from the lower half of the normal weight range to the upper half of the overweight range. The key finding, however, is the greater risk of being classified in the ‘increasing’ and ‘highest’ trajectory classes in individuals born in the most recent cohort (1970 BCS) compared to those born in the 1946 NSHD. Whilst an increased risk of being in the ‘highest’ trajectory class was also observed for those in the 1958 NCDS (relative to those in 1946 NSHD), there was no increased risk of being classified in the ‘increasing’ trajectory for those in this cohort.

The major novelty and most substantial contribution our study makes to the literature comes from the identification of how risk for displaying certain trajectories of BMI development differs between distinct cohorts born over a 24-year period. Evidence of a skewing of the distribution was apparent even in the 1958 NCDS cohort, as evidenced by the increased risk of being in the ‘highest’ trajectory class compared to those in the 1946 NSHD. However, only in the most recent cohort was there an increased risk of displaying the trajectory characterised by rapid increases in BMI which resulted in the crossing of several BMI categories. We therefore provide empirical evidence that increases in the prevalence of adult overweight and obesity observed in more recent cohorts are not simply a result of the skewing of the BMI distribution but also by altered patterns of BMI development, namely a greater likelihood for displaying a trajectory characterised by rapid increases in BMI over the life course.

Our work supports and extends previous BMI secular trend research conducted in these cohorts using different analytical approaches [2, 25, 26]. For example, Li et al. [25] used multilevel models (MLMs) to compare life course BMI trajectories for those in the 1946 NSHD and 1958 NCDS. They observed that compared to individuals in the 1946 NSHD, individuals in the 1958 NCDS gained BMI more rapidly after early adulthood so that by mid-adulthood, individuals in the 1958 NCDS had a larger BMI by 1–2 kg/m^2^. We extend this work in two ways. Firstly, we have demonstrated secular changes in BMI trajectories with the inclusion of the 1970 BCS and thus a cohort of individuals, who unlike the other two studies, experienced the obesity epidemic from childhood. Secondly, unlike the MLM approach, by using mixture models, we have been able to classify individuals into discrete BMI trajectory sub-populations and subsequently identify how birth year contributes to the probability of being assigned to a particular class. To our knowledge, Sperrin and colleagues [8] were the first to illustrate secular trends in BMI development using mixture models. Using annual cross-sectional surveys of the Health Survey for England between 1992 and 2010, they identified a normal BMI ‘resistant’ sub-population in which mean BMI remained relatively stable and a high BMI ‘fatter’ sub-population getting ‘fatter’. However, the use of repeated cross-sectional surveys as a proxy for longitudinal data meant the authors were unable to identify latent classes of individuals who share similar patterns of BMI development over age and document how birth year was related to the probability of being in each class. Furthermore, as only trends in BMI from early adulthood onwards were studied, the study is unable to provide insight into how the process of obesity develops across the life course. More recently, Nedelec and colleagues [9] pooled serial BMI data between 2 and 20 years in two separate Finnish cohorts, one born in 1966 and the other in 1986, with the aim of investigating generational effects on the development of BMI trajectories. As was the case in our study, the authors identified four sub-populations of individuals, with the average BMI trajectories of the four sub-populations characterised as persistently below average weight, persistently average weight, persistently above average weight, and persistently large. The observation of a persistently large sub-population is in line with our findings and is indicative of a right-skewing of the BMI distribution. However, whereas we observed a greater likelihood of being assigned to this class in the most recent cohort, in the study by Nedelec and colleagues, there was no difference in the proportions of the two cohorts assigned to this class. In addition, they did not observe the rapidly increasing trajectory which we identified, which saw individuals transition from a healthy normal weight to the upper-end of the overweight range. The reasons for this are unclear and could relate to differences in the modelling approach, the cohorts used (age and interval) or the follow-up period. Alternatively, it may be the case that no secular trend in BMI development has occurred in this population, which could be a consequence of a historically less obesogenic environment in Finland compared to the UK, evidenced by the lower Finnish obesity rates observed over time [1].

We are not aware of any secular trend research which has documented how cohorts exposed to the obesogenic environment for varying periods of their life course, contribute differently not only to the skewing of the BMI distribution, but also to changes in the pattern of BMI development over age. As such, we are the first to document an increased likelihood of being assigned to a sub-population displaying a trajectory characterised by rapid increases in BMI over the life course in those with more prolonged exposure to the obesogenic environment. However, whilst those in the most recent cohorts showed an increased likelihood of being assigned to this sub-population, we still observed over 20% of the 1946 NSHD cohort being assigned to this sub-population, suggesting that this is an important sub-population of BMI development not exclusively demonstrated by more recent cohorts, but which is likely to become more prevalent with time. Given the increased prevalence of adverse cardiometabolic outcomes observed in individuals who display this pattern of BMI development [11], a greater focus on identifying this pattern of BMI gain, during any stage of the life course and at the individual or population level, is required. For example, current clinical guidelines in the UK recommend using ‘clinical judgement’ to decide when to measure a person’s BMI [27]. As we have demonstrated, however, many individuals demonstrating this ‘increasing’ BMI trajectory are likely to remain in the normal weight BMI category for a period of time and thus there may not appear to be any indication for measuring BMI in clinic. As such, to identify this pattern of BMI development in a timely manner, we advocate regular routine BMI measurements to be taken, especially during adolescence, when BMI gain in this sub-population is highest.

We observed that the ‘increasing’ and ‘highest’ BMI trajectories were more common in females (37.9%) than males (26.2%) which is in line with current estimates of obesity in the UK, which reveal a greater obesity burden in females [28]. Potential explanations for this could be biological, e.g. the differing hormonal profile between the sexes may interact with an increasingly obesogenic environment and/or sociocultural, e.g. a differential exposure obesogenic environments [29].

The key strength of our study is the identification, using approximately 104,000 serial BMI observations spanning childhood to adulthood, of meaningful life course BMI trajectory classes. These trajectory classes were identified after a detailed model specification process which considered several age functions for the trajectory shape, removal of default constraints on the growth term variances and covariances, different specifications of the within-class residual variance/error structure, and different autocorrelation structures. In particular, our growth mixture models allowed within-class variance to be freely estimated, meaning individuals within a class were allowed to display different trajectories. This is in contrast to another type of mixture model commonly used to identify distinct groups of individuals with similar trajectories [9, 30], namely, group-based trajectory models (also known as latent class trajectory models). In these models, within-class variability is fixed to zero, meaning all individuals within a class display the same trajectory. This assumption is unrealistic and often results in poorly fitting models which violate key assumptions such as serial independence of residuals. We acknowledge several limitations. As a consequence of our desire to capture each individual’s growth trajectory as accurately as possible, we enabled individuals to vary from the average trajectory of their class. A consequence of this is a reduction in our relative entropy indicating how well an individual fits into each class. However, as per the expert guidance on such models [19], the BIC, rather than entropy, should be used to determine the optimum number of classes, which is the approach we adopted. The cohorts used in this study are of individuals born between 1946 and 1970, given the increased exposure to and severity of the obesogenic environment experienced today, if repeated with a younger cohort, we may have observed a greater number individuals in the ‘increasing’ or ‘highest’ BMI trajectories. Alternatively, we may have observed more extreme trajectories (i.e. greater increases in the ‘increasing’ class or a higher trajectory in the ‘highest’ class). This work was based on the pooling of data from three cohort studies which requires the collection of the same (or harmonised) variables in each of the cohorts. Measurement protocols for weight and height were not consistent within and between studies, with more self-reported weight and height data in the BCS70. This may have introduced bias if self-reported measurements were systemically under or over-reported. For example, it has been shown that people with greater BMIs tend to under-report their weight [31, 32]. Finally, a pragmatic decision was made to limit the sample to those with at least BMI measurements in order to aid classification. By doing this, we have selected a sample which was more likely to be White British and with more educated parents. This may limit the generalisability of our findings.

## Conclusion

Our results suggest that the obesity epidemic in Great Britain reflects not only an upward shift in BMI trajectories but also a more recent increase in the number of individuals demonstrating more rapid weight gain, from normal weight to overweight, across the second, third, and fourth decades of life. Given that contemporaneous cohorts are likely to experience an even more severe and protracted exposure to obesogenic environments, it is likely that this pattern of BMI development is likely to increase either in terms of the number of the population displaying it or in the severity of the gains in BMI observed.

## Supplementary Information

**Additional file 1: Text S1.** Mixture modelling. **Table S1.** Description of the longitudinal anthropometric data: males. **Table S2.** Description of the longitudinal anthropometric data: females**. Table S3.** Characteristics of those included in the final sample (*n* = 25 655) vs those excluded for having less than 3 BMI measurements (*n* = 10 538). **Table S4.** Comparison of the BIC between mixture models (1–7 classes) with different specifications: males**. Table S5.** Comparison of the BIC between mixture models (1–7 classes) with different specifications: females. **Table S6.** Summary of final mixture models (1–7 classes): males. **Table S7.** Summary of final mixture models (1–7 classes): females. **Figure S1.** Distribution of posterior probabilities for assigned class membership for the selected 4-class model: males. **Figure S2.** Distribution of posterior probabilities for assigned class membership for the selected 4-class model: females. **Table S8.** Descriptive statistics for each BMI trajectory latent class: males (*n* = 12 465). **Table S9.** Descriptive statistics for each BMI trajectory latent class: females (*n* = 13 190).

**Additional file 2: Text S1.** Mplus output file: males. **Text S2.** Mplus output file: females.

## Data Availability

1946 NSHD data are available from https://www.nshd.mrc.ac.uk/data/data-sharing/; 1958 NCDS and 1970 BCS data are available from the UK Data Archive: https://www.data-archive.ac.uk.

## Abbreviations

AR1: First-order autoregressive structure
BCS: British Cohort Study
BIC: Bayesian Information criterion
BMI: Body mass index
IOTF: International Obesity Task Force
NCDS: National Child Development Study
NSHD: National Survey of Health and Development
OR: Odds ratio

## References

[1] Non-Communicable Diseases Risk Factor Collaboration (2017). Worldwide trends in body-mass index, underweight, overweight, and obesity from 1975 to 2016: a pooled analysis of 2416 population-based measurement studies in 128·9 million children, adolescents, and adults. Lancet..

[2] Johnson W, Li L, Kuh D, Hardy R. How has the age-related process of overweight or obesity development changed over time? Co-ordinated analyses of individual participant data from five United Kingdom birth cohorts. Plos Med. 2015;12(5). 10.1371/journal.pmed.1001828. 10.1371/journal.pmed.1001828 PMC4437909 25993005

[3] Flegal KM, Troiano RP (2000). Changes in the distribution of body mass index of adults and children in the US population. Int J Obes Relat Metab Disord.

[4] Walls HL, Wolfe R, Haby MM, Magliano DJ, De Courten M, Reid CM (2010). Trends in BMI of urban Australian adults, 1980-2000. Public Health Nutr.

[5] Razak F, Corsi DJ, SV Subramanian S. (2013). Change in the body mass index distribution for women: analysis of surveys from 37 low- and middle-income countries. Plos Med.

[6] Hayes A, Gearon E, Backholer K, Bauman A, Peeters A (2015). Age-specific changes in BMI and BMI distribution among Australian adults using cross-sectional surveys from 1980 to 2008. Int J Obes.

[7] Ravussin E, Bouchard C (2000). Human genomics and obesity: finding appropriate drug targets. Eur J Pharmacol.

[8] Sperrin M, Marshall AD, Higgins V, Buchan IE, Renehan AG (2014). Slowing down of adult body mass index trend increases in England: a latent class analysis of cross-sectional surveys (1992-2010). Int J Obes.

[9] Nedelec R, Miettunen J, Männikkö M, Järvelin MR, Sebert S (2020). Maternal and infant prediction of the child BMI trajectories; studies across two generations of Northern Finland birth cohorts. Int J Obes.

[10] Di Cesare M, Sorić M, Bovet P, Miranda JJ, Bhutta Z, Stevens GA (2019). The epidemiological burden of obesity in childhood: a worldwide epidemic requiring urgent action. BMC Med.

[11] Ohlsson C, Bygdell M, Sonden A, Rosengren A, Kindblom JM (2016). Association between excessive BMI increase during puberty and risk of cardiovascular mortality in adult men: a population-based cohort study. Lancet Diabetes Endocrinol.

[12] Wadsworth M, Kuh D, Richards M, Hardy R (2006). Cohort profile: the 1946 National Birth Cohort (MRC National Survey of Health and Development). Int J Epidemiol.

[13] Power C, Elliott J (2006). Cohort profile: 1958 British birth cohort (National Child Development Study). Int J Epidemiol.

[14] Elliott J, Shepherd P (2006). Cohort profile: 1970 British Birth Cohort (BCS70). Int J Epidemiol.

[15] Kuh D, Pierce M, Adams J, Deanfield J, Ekelund U, Friberg P, Ghosh AK, Harwood N, Hughes A, Macfarlane PW, Mishra G, Pellerin D, Wong A, Stephen AM, Richards M, Hardy R, on behalf of the NSHD scientific and data collection team (2011). Cohort profile: updating the cohort profile for the MRC National Survey of Health and Development: a new clinic-based data collection for ageing research. Int J Epidemiol.

[16] Muthén B, Shedden K (1999). Finite mixture modeling with mixture outcomes using the EM algorithm. Biometrics..

[17] Brault MC, Aimé A, Bégin C, Valois P, Craig W (2015). Heterogeneity of sex-stratified BMI trajectories in children from 8 to 14years old. Physiol Behav.

[18] Oluwagbemigun K, Buyken AE, Alexy U, Schmid M, Herder C, Nöthlings U (2019). Developmental trajectories of body mass index from childhood into late adolescence and subsequent late adolescence-young adulthood cardiometabolic risk markers. Cardiovasc Diabetol.

[19] van de Schoot R, Sijbrandij M, Winter SD, Depaoli S, Vermunt JK (2017). The GRoLTS-Checklist: guidelines for reporting on latent trajectory studies. Struct Equ Model A Multidiscip J.

[20] Schwarz GE (1978). Estimating the dimension of a model. Ann Stat.

[21] Li S, Chen W, Sun D, Fernandez C, Li J, Kelly T, He J, Krousel-Wood M, Whelton PK (2015). Variability and rapid increase in body mass index during childhood are associated with adult obesity. Int J Epidemiol.

[22] Gilthorpe MS, Dahly DL, Tu YK, Kubzansky LD, Goodman E (2014). Challenges in modelling the random structure correctly in growth mixture models and the impact this has on model mixtures. J Dev Orig Heal Dis.

[23] Cole TJ, Lobstein T (2012). Extended international (IOTF) body mass index cut-offs for thinness, overweight and obesity. Pediatr Obes.

[24] Vermunt JK (2010). Latent class modeling with covariates: two improved three-step approaches. Polit Anal.

[25] Li L, Hardy R, Kuh D, Lo Conte R, Power C (2008). Child-to-adult body mass index and height trajectories: a comparison of 2 British birth cohorts. Am J Epidemiol.

[26] Li L, Hardy R, Kuh D, Power C (2015). Life-course body mass index trajectories and blood pressure in mid life in two British birth cohorts: stronger associations in the later-born generation. Int J Epidemiol.

[27] Excellence NI for H and C. Obesity: identification, assessment and management. London, UK; 2014. nice.org.uk/guidance/cg189.

[28] Lifestyles team ND. Statistics on obesity, Physical Activity and Diet, England, 2020. 2020. https://digital.nhs.uk/data-and-information/publications/statistical/statistics-on-obesity-physical-activity-and-diet/england-2020/part-3-adult-obesity-copy. Accessed 8 Mar 2021.

[29] Sweeting HN. Gendered dimensions of obesity in childhood and adolescence. Nutr J. 2008;7(1). 10.1186/1475-2891-7-1. 10.1186/1475-2891-7-1 PMC2265740 18194542

[30] Mattsson M, Maher GM, Boland F, Fitzgerald AP, Murray DM, Biesma R (2019). Group-based trajectory modelling for BMI trajectories in childhood: a systematic review. Obes Rev.

[31] Crawley HF, Portides G (1995). Self-reported versus measured height, weight and body mass index amongst 16-17 year old British teenagers. Int J Obes Relat Metab Disord.

[32] Shields M, Gorber SC, Tremblay MS (2008). Estimates of obesity based on self-report versus direct measures. Heal Reports.

