# Supplementary material for: Changes over time in latent patterns of childhood-to-adulthood BMI development in Great Britain: evidence from three cohorts born in 1946, 1958, and 1970
Source: BMC Med. 2021 Apr 21;19:96. doi: 10.1186/s12916-021-01969-8 (PMC8059270; doi:10.1186/s12916-021-01969-8)
Supplement: Supplementary file 1 — Additional file 1: Text S1. Mixture modelling. Table S1. Description of the longitudinal anthropometric data: males. Table S2. Description of the longitudinal anthropometric data: females. Table S3. Characteristics of those included in the final sample (n = 25 655) vs those excluded for having less than 3 BMI measurements (n = 10 538). Table S4. Comparison of the BIC between mixture models (1–7 classes) with different specifications: males. Table S5. Comparison of the BIC between mixture models (1–7 classes) with different specifications: females. Table S6. Summary of final mixture models (1–7 classes): males. Table S7. Summary of final mixture models (1–7 classes): females. Figure S1. Distribution of posterior probabilities for assigned class membership for the selected 4-class model: males. Figure S2. Distribution of posterior probabilities for assigned class membership for the selected 4-class model: females. Table S8. Descriptive statistics for each BMI trajectory latent class: males (n = 12 465). Table S9. Descriptive statistics for each BMI trajectory latent class: females (n = 13 190). [file 12916_2021_1969_MOESM1_ESM.docx]

**Text S1: Mixture modelling**

We used Mplus (version 8.3) to develop a single growth mixture model that identified distinct groups of individuals who had similar childhood-adulthood BMI trajectories between 11-42 years of age.

BMI was assessed at all sweeps used in the present paper. Mplus requires the data to be in wide format, with a separate column for BMI data at each sweep. As described in the methods, we dropped individuals who did not have three or more serial BMI measurements. This reduced the sample size by 10538 and the number of BMI observations by 16597. Tables S1 and S2 provide a detailed description of the sweeps and anthropometric data used for analysis.

For 1-7 class solutions, we fitted a series of mixture models in which the longitudinal BMI response was described using each of the following age functions: linear, freed-loading, quadratic, and cubic polynomial. With the freed-loading function, one factor loading is set to zero, another to one, and the rest are freely estimated, resulting in a type of non-linear “spline” which flexibly fits the data between adjacent time points (1).. Because outcome variances were not consistent across the sweeps, we did not consider constraining the BMI residual variances (i.e., errors) to be identical across sweeps (i.e., heteroskedasticity was assumed). Using the cubic polynomial models, we next tried to relax some of the main default constraints implemented by Mplus. Allowing the residual variances/errors to differ across the classes improved model fit by between 3748 and 5324 BIC points in males and 4928 and 6019 in females. We then attempted to extend the models to include a within-class autocorrelation structure for the residual variances/errors. We started with a full autoregressive correlation model (AR (1)) and after this did not converge, we fitted decreasingly complex correlation structures until converge was achieved. Final models included within class regressions of BMI_t_ on BMI_t-1_. This improved model fit by between 63 and 1084 BIC points in males and 2722 and 5313 in females. Finally, because between-individual variation in BMI is theoretically greater among obese compared to normal-weight children (2,3), we tested models that allowed the variance of the latent intercept of the growth curve function to differ between classes, but none of the models would converge.

This model was run for 1-7 class solutions. To avoid convergence at local minima (4), 1000 random starts (for 20 iterations) were preformed, of which the best 200 models (according to log-likelihood) were run to completion (STARTS = 1000 200; STITERATIONS = 20). In all instances, the best log-likelihood was replicated. A summary of the final mixture models (1-7 classes), including measures of class separation, is presented in Tables S7 and S8.

Our choice of which of the 1-7 class solutions to select for further investigation was based on, firstly, overall fit according to the BIC and, secondly, interpretability of the average trajectories.6 As shown in Tables S7 and S8, the reduction in BIC between k and k – 1 solutions attenuated exponentially as the number of classes increased.

In both males and females, we felt that by the 5-class solution the reduction in BIC (e.g., compared to the 4-class solution) was becoming less important. For this reason, we now focus on discussing the choice between the 4-class and 5-class solutions. The entropy was slightly higher (i.e., better) in the 4-class solution, while the average posterior probabilities of class membership were always greater than the proposed cut-off of 0.65 in the 4-class solution but not in the 5-class solution (5). All classes in the 5-class solution comprised a reasonable number of participants, with the lowest proportion of individuals being 5.74% (males, class 1). In the 5-class solution however, the smallest proportion was <2% (males, class 1). We therefore selected the 4-class solution as our final model. Four further figures were produced. For each sex, a figure showing the distribution of posterior probabilities for assigned class membership (Figure S1 and S2) and another showing the final fitted trajectories for each class (Figures 1 and 2).

**References**

1. Bollen KA, Curran PJ. Latent Curve Models [Internet]. Latent Curve Models: A Structural Equation Perspective. Hoboken, NJ, USA: John Wiley & Sons, Inc.; 2005 [cited 2020 Dec 2]. 1-293 p. (Wiley Series in Probability and Statistics). Available from: http://doi.wiley.com/10.1002/0471746096

2. Flegal KM, Troiano RP. Changes in the distribution of body mass index of adults and children in the US population. Int J Obes Relat Metab Disord [Internet]. 2000;24(7):807–18. Available from: http://www.ncbi.nlm.nih.gov/pubmed/10918526

3. Johnson W, Li L, Kuh D, Hardy R. How Has the Age-Related Process of Overweight or Obesity Development Changed over Time? Co-ordinated Analyses of Individual Participant Data from Five United Kingdom Birth Cohorts. PLoS Med. Public Library of Science; 2015 May 1;12(5).

4. Hipp JR, Bauer DJ. Local solutions in the estimation of growth mixture models. Psychol Methods [Internet]. 2006;11(1):36–53. Available from: http://www.ncbi.nlm.nih.gov/pubmed/16594766

5. Marioni RE, Proust-Lima C, Amieva H, Brayne C, Matthews FE, Dartigues JF, et al. Cognitive lifestyle jointly predicts longitudinal cognitive decline and mortality risk. Eur J Epidemiol [Internet]. 2014;29(3):211–9. Available from: http://www.ncbi.nlm.nih.gov/pubmed/24577561

**Table S1. Description of the longitudinal anthropometric data: males**

| **Visit** | **Age (years)** | | | | | | **BMI (kg/m^2^)** | | | |
| --- | --- | --- | --- | --- | --- | --- | --- | --- | --- | --- |
|  | **25^th^ centile** | **50^th^ centile** | **75^th^ centile** | **Min** | **Max** | **Range** | **N** | **50^th^ centile** | **25^th^ centile** | **75^th^ centile** |
| 1 | 10.31 | 11.04 | 11.33 | 9.79 | 12.84 | 3.05 | 10707 | 16.69 | 15.68 | 18.01 |
| 2 | 15.65 | 15.84 | 16.28 | 14.33 | 17.61 | 3.28 | 9279 | 19.93 | 18.55 | 21.61 |
| 3 | 23.54 | 23.64 | 26.00 | 22.80 | 27.42 | 4.62 | 9341 | 23.09 | 21.47 | 24.94 |
| 4 | 33.29 | 33.93 | 34.36 | 32.90 | 37.08 | 4.19 | 10350 | 25.26 | 23.23 | 27.71 |
| 5 | 41.92 | 42.19 | 42.60 | 41.32 | 44.33 | 3.02 | 10174 | 26.11 | 23.98 | 28.74 |

**Table S2. Description of the longitudinal anthropometric data: females**

| **Visit** | **Age (years)** | | | | | | **BMI (kg/m^2^)** | | | |
| --- | --- | --- | --- | --- | --- | --- | --- | --- | --- | --- |
|  | **25^th^ centile** | **50^th^ centile** | **75^th^ centile** | **Min** | **Max** | **Range** | **N** | **50^th^ centile** | **25^th^ centile** | **75^th^ centile** |
| 1 | 10.28 | 10.92 | 11.33 | 9.73 | 12.67 | 2.94 | 11248 | 16.88 | 15.63 | 18.58 |
| 2 | 15.66 | 15.88 | 16.30 | 14.33 | 18.52 | 4.19 | 9877 | 20.64 | 19.05 | 22.57 |
| 3 | 23.54 | 26.00 | 26.00 | 22.80 | 27.42 | 4.61 | 11399 | 21.87 | 20.34 | 24.02 |
| 4 | 33.28 | 33.94 | 34.35 | 32.99 | 36.92 | 3.93 | 10887 | 23.46 | 21.41 | 26.50 |
| 5 | 41.92 | 42.19 | 42.60 | 41.29 | 44.42 | 3.13 | 10667 | 24.41 | 22.13 | 27.92 |

**Table S3** **Characteristics of those included in the final sample (n=25 655) vs those excluded for having less than 3 BMI measurements (n=10 538)**

*Original values (i.e., not adjusted for medication use)

|  | **Included in final sample (n=25 655)** | | **Excluded as <3 BMI measurements (n=10 538)** | | ***p* for diff*** |
| --- | --- | --- | --- | --- | --- |
|  | *total n* |  | *total n* |  |  |
|  |  | | | | |
| Sex (male, n, (%)) | 25 655 | 12 465 (48.6) | 10 538 | 6 113 (58.0) | p<0.001 |
| Birth weight (kg, mean (SD)) | 23 969 | 3.34 (0.5) | 9 331 | 3.30 (0.5) | P=0.004 |
| Ethnicity (White British, n (%)) | 25 552 | 24 884 (97.4) | 8 848 | 8 361 (94.5) | p<0.001 |
| Mother left education at mandatory leaving age (yes, n (%)) | 24 267 | 16 862 (69.5) | 9 384 | 6 948 (74.0) | p<0.001 |
| Father left education at mandatory leaving age (yes, n (%)) | 22 700 | 15 744 (69.4) | 7 713 | 5 642 (73.2) | p<0.001 |
| Father occupational Social class (Professional or Management, n (%))) | 22 624 | 6 222 (27.5) | 7 537 | 1 658 (22.0) | p<0.001 |

**Table S4. Comparison of the BIC between mixture models (1-7 classes) with different specifications: males**

|  | **Model 1** | **Model 2** | | **Model 3** | | **Model 4** | | **Model 5** | | **Model 6** | |
| --- | --- | --- | --- | --- | --- | --- | --- | --- | --- | --- | --- |
|  | Linear | Quadratic  polynomial | | Cubic  polynomial^a^ | | Free loading | | Model 3 + Residual variances  (of BMI measures)  allowed to differ  across classes | | Model 5 + inclusion autoregression structure^b^ | |
|  |  |  | Δ from  model 1 |  | Δ from  model 2 |  | Δ from  model 3 |  | Δ from  model 3 |  | Δ from  model 5 |
| Class |  |  |  |  |  |  |  |  |  |  |  |
| 1 | 241358 | 227683 | -13675 | 227552 | -131 | 227490 | -62 | 227671 | +119 | 227608 | -63 |
| 2 | 239388 | 225130 | -14258 | 225624 | +494 | 225012 | -612 | 220300 | -5324 | 219010 | -1290 |
| 3 | 238434 | 224019 | -14415 | 223633 | -386 | 223913 | +280 | 219322 | -4311 | 218248 | -1084 |
| 4 | 238105 | 223555 | -14550 | 223014 | -541 | 223523 | +509 | 218622 | -4392 | 217748 | -874 |
| 5 | 237847 | 223178 | -14669 | 222524 | -654 | 223268 | +744 | 218296 | -4228 | 217655 | -641 |
| 6 | 237673 | 222895 | -14778 | 222056 | -839 | 223080 | +1024 | 218132 | -3924 | 217628 | -504 |
| 7 | 237579 | 222721 | -14858 | 221755 | -966 | 222909 | +1154 | 218007 | -3748 | 217608 | -399 |

^a^Variances of the slope 2 (x2) and 3 terms (x3) and all covariances involving these terms had to be constrained to be zero for models to converge without error messages ^b^autoregression of adjacent BMI measurements

**Table S5. Comparison of the BIC between mixture models (1-7 classes) with different specifications: females**

|  | **Model 1** | **Model 2** | | **Model 3** | | **Model 4** | | **Model 5** | | **Model 6** | |
| --- | --- | --- | --- | --- | --- | --- | --- | --- | --- | --- | --- |
|  | Linear | Quadratic  polynomial | | Cubic  polynomial^a^ | | Free loading | | Model 3 + Residual variances  (of BMI measures)  allowed to differ  across classes | | Model 5 + inclusion autoregression structure^b^ | |
|  |  |  | Δ from  model 1 |  | Δ from  model 2 |  | Δ from  model 3 |  | Δ from  model 3 |  | Δ from  model 5 |
| Class |  |  |  |  |  |  |  |  |  |  |  |
| 1 | 273551 | 266209 | -7342 | 264390 | -1819 | 264161 | -229 | 265485 | +1095 | 262763 | -2722 |
| 2 | 270544 | 262978 | -7566 | 260622 | -2356 | 261073 | +451 | 255694 | -4928 | 250381 | -5313 |
| 3 | 269466 | 261689 | -7757 | 259515 | -2174 | 259851 | +336 | 253496 | -6019 | 248803 | -4693 |
| 4 | 268848 | 260596 | -8252 | 258113 | -2483 | 259297 | +1184 | 252511 | -5602 | 248039 | -4472 |
| 5 | 268391* | 260060 | -8331 | 257619 | -2441 | 258672 | +1053 | 252015 | -5604 | 247818 | -4197 |
| 6 | 268102* | 259597 | -8505 | 257126 | -2471 | 258447 | +1321 | 251730 | -5396 | 247661 | -4069 |
| 7 | 267969* | 259273 | -8696 | 256645 | -2628 | 258247 | +1602 | 251503 | -5142 | 247668 | -3835 |

**Table S6. Summary of final mixture models (1-7 classes): males**

| **Classes** | **1** | **2** | **3** | **4** | **5** | **6** | **7** |
| --- | --- | --- | --- | --- | --- | --- | --- |
| AIC | 227504 | 218787 | 217921 | 217317 | 217120 | 216989 | 216865 |
| BIC | 227608 | 219010 | 218248 | 217748 | 217655 | 217628 | 217608 |
| BIC difference | -- | 8598 | 762 | 500 | 93 | 27 | 20 |
| BLRT p-value | -- |  |  |  |  |  |  |
| Entropy | -- | 0.70 | 0.57 | 0.51 | 0.50 | 0.46 | 0.50 |
| Posterior probability [mean] |  |  |  |  |  |  |  |
| Class-1 | -- | 0.88 | 0.85 | 0.84 | 0.81 | 0.60 | 0.65 |
| Class-2 | -- | 0.93 | 0.76 | 0.75 | 0.62 | 0.82 | 0.60 |
| Class-3 | -- |  | 0.81 | 0.69 | 0.69 | 0.58 | 0.73 |
| Class-4 | -- |  |  | 0.68 | 0.67 | 0.60 | 0.82 |
| Class-5 | -- |  |  |  | 0.71 | 0.72 | 0.58 |
| Class-6 | -- |  |  |  |  | 0.64 | 0.84 |
| Class-7 | -- |  |  |  |  |  | 0.60 |
| Posterior probability > 0·7 [%] |  |  |  |  |  |  |  |
| Class-1 | -- | 81.79 | 76.66 | 75.14 | 70.29 | 29.84 | 39.59 |
| Class-2 | -- | 94.21 | 66.41 | 65.88 | 31.35 | 71.17 | 29.80 |
| Class-3 | -- |  | 77.95 | 49.01 | 51.95 | 21.40 | 56.08 |
| Class-4 | -- |  |  | 45.64 | 45.08 | 26.47 | 72.67 |
| Class-5 | -- |  |  |  | 55.25 | 55.87 | 21.50 |
| Class-6 | -- |  |  |  |  | 37.42 | 78.00 |
| Class-7 | -- |  |  |  |  |  | 25.03 |
| Class membership [N (%)] |  |  |  |  |  |  |  |
| Class-1 | (100.00) | 2339 (18.80) | 767 (6.15) | 716 (5.74) | 239 (1.92) | 2926 (23.47) | 1283 (10.29) |
| Class-2 |  | 10126 (81.20) | 4504 (36.13) | 7160 (57.44) | 2373 (19.04) | 163 (1.31) | 2960 (23.75) |
| Class-3 |  |  | 7194 (57.71) | 2030 (16.29) | 6337 (50.84) | 3440 (27.60) | 576 (4.62) |
| Class-4 |  |  |  | 2559 (20.53) | 2611 (20.95) | 4065 (32.61) | 150 (1.20) |
| Class-5 |  |  |  |  | 905 (7.26) | 639 (5.13) | 3530 (28.32) |
| Class-6 |  |  |  |  |  | 1232 (9.88) | 50 (0.40) |
| Class-7 |  |  |  |  |  |  | 3916 (31.42) |

**Table S7. Summary of final mixture models (1-7 classes): females**

| **Classes** | **1** | **2** | **3** | **4** | **5** | **6** | **7** |
| --- | --- | --- | --- | --- | --- | --- | --- |
| AIC | 262658 | 250156 | 248474 | 247605 | 247279 | 247017 | 246919 |
| BIC | 262763 | 250381 | 248803 | 248039 | 247818 | 247661 | 247668 |
| BIC difference | -- | 12382 | 1578 | 764 | 221 | 157 | +7 |
| BLRT p-value | -- |  |  |  |  |  |  |
| Entropy | -- | 0.7 | 0.61 | 0.57 | 0.52 | 0.51 | 0.51 |
| Posterior probability [mean] |  |  |  |  |  |  |  |
| Class-1 | -- | 0.91 | 0.83 | 0.70 | 0.69 | 0.69 | 0.62 |
| Class-2 | -- | 0.92 | 0.87 | 0.74 | 0.67 | 0.63 | 0.68 |
| Class-3 | -- |  | 0.80 | 0.78 | 0.73 | 0.62 | 0.69 |
| Class-4 | -- |  |  | 0.86 | 0.85 | 0.82 | 0.58 |
| Class-5 | -- |  |  |  | 0.62 | 0.66 | 0.81 |
| Class-6 | -- |  |  |  |  | 0.66 | 0.63 |
| Class-7 | -- |  |  |  |  |  | 0.56 |
| Posterior probability > 0·7 [%] |  |  |  |  |  |  |  |
| Class-1 | -- | 87.49 | 80.84 | 46.96 | 50.45 | 50.15 | 32.04 |
| Class-2 | -- | 92.84 | 82.25 | 60.41 | 43.73 | 36.99 | 45.82 |
| Class-3 | -- |  | 75.08 | 71.46 | 59.27 | 31.58 | 49.93 |
| Class-4 | -- |  |  | 78.65 | 76.75 | 72.48 | 22.94 |
| Class-5 | -- |  |  |  | 31.06 | 43.95 | 67.37 |
| Class-6 | -- |  |  |  |  | 44.05 | 37.20 |
| Class-7 | -- |  |  |  |  |  | 13.39 |
| Class membership [N (%)] |  |  |  |  |  |  |  |
| Class-1 | (100.0) | 3661 (27.76) | 6191 (46.94) | 1942 (14.72) | 4581 (34.73) | 1689 (12.81) | 2990 (22.67) |
| Class-2 |  | 9529 (72.24) | 1442 (10.93) | 3771 (28.59) | 2161 (16.38) | 2441 (18.51) | 1185 (8.98) |
| Class-3 |  |  | 5557 (42.13) | 6250 (47.38) | 2504 (18.98) | 3857 (29.24) | 763 (5.78) |
| Class-4 |  |  |  | 1227 (9.30) | 744 (5.64) | 447 (3.39) | 3191 (24.19) |
| Class-5 |  |  |  |  | 3200 (24.26) | 1140 (8.64) | 331 (2.51) |
| Class-6 |  |  |  |  |  | 3616 (27.41) | 2586 (19.61) |
| Class-7 |  |  |  |  |  |  | 2144 (16.25) |

**Figure S1. Distribution of posterior probabilities for assigned class membership for the selected 4-class model: males
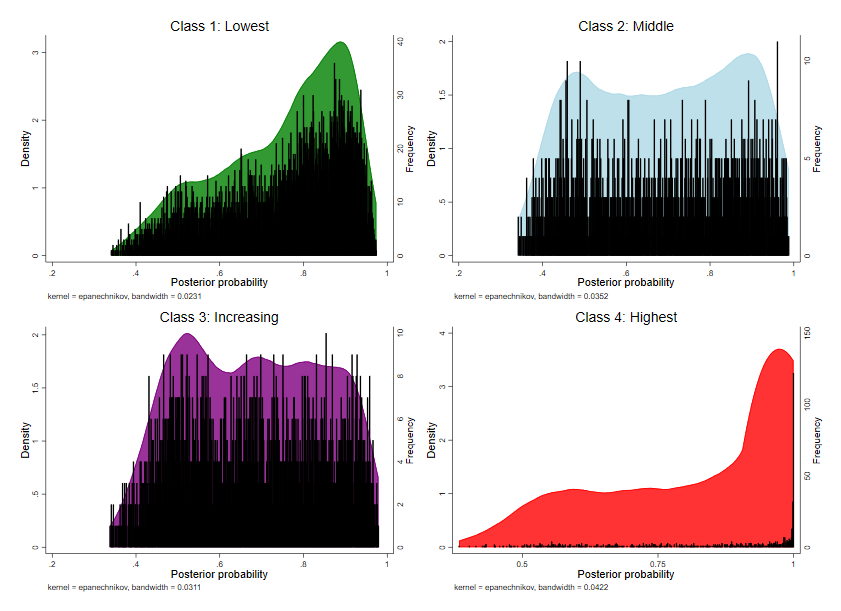
**

**Figure S2. Distribution of posterior probabilities for assigned class membership for the selected 4-class model: females**

**
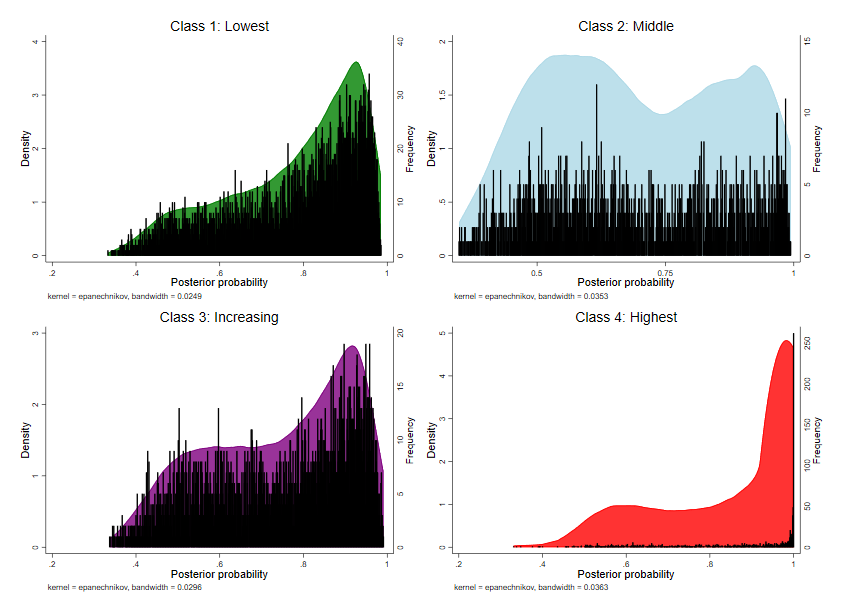
**

**Table S8. Descriptive statistics for each BMI trajectory latent class: males (n= 12 465)**

|  |  |  |  |  |  |
| --- | --- | --- | --- | --- | --- |
|  |  | *Class 1* | *Class 2* | *Class 3* | *Class 4* |
|  |  | *Lowest* | *Middle* | *Increasing* | *Highest* |
| **Ethnicity** |  |  |  |  |  |
| White British | n (%) | 6960 (97.6) | 1974 (97.5) | 2473 (96.9) | 688 (96.8) |
| Other^a^ | n (%) | 174 (2.4) | 51 (2.5) | 78 (3.1) | 23 (3.2) |
| **Birthweight (kg)** | mean (SD) | 3.4 (0.5) | 3.5 (0.5) | 3.4 (0.5) | 3.4 (0.5) |
| **Childhood social class** |  |  |  |  |  |
| Professional | n (%) | 394 (6.3) | 126 (6.9) | 76 (3.4) | 22 (3.5) |
| Intermediate | n (%) | 1449 (23.0) | 439 (24.0) | 419 (18.5) | 122 (19.4) |
| Skilled non-manual | n (%) | 800 (12.7) | 219 (12.0) | 210 (9.3) | 66 (10.5) |
| Skilled manual | n (%) | 2359 (37.4) | 697 (38.1) | 984 (43.5) | 282 (44.9) |
| Partly skilled manual | n (%) | 879 (13.9) | 224 (12.3) | 373 (16.5) | 95 (15.1) |
| Unskilled manual | n (%) | 424 (6.7) | 123 (6.7) | 201 (8.9) | 41 (6.5) |
| **Mother left education at mandatory leaving age** |  |  |  |  |  |
| *Yes* | n (%) | 4563 (67.6) | 1308 (68.3) | 1791 (74.0) | 532 (78.0) |
| **Father left education at mandatory leaving age** |  |  |  |  |  |
| *Yes* | n (%) | 4366 (68.1) | 1206 (66.7) | 1658 (74.2) | 513 (79.7) |

^a^Other ethnicities: White other, Mixed race, Indian, Pakistani, Bangladeshi, Other Asian, Caribbean, African, Other Black, Chinese

**Table S9. Descriptive statistics for each BMI trajectory latent class: females (n=13 190)**

|  |  |  |  |  |  |
| --- | --- | --- | --- | --- | --- |
|  |  | *Class 3* | *Class 1* | *Class 2* | *Class 4* |
|  |  | *Lowest* | *Middle* | *Increasing* | *Highest* |
| **Ethnicity** |  |  |  |  |  |
| White British | n (%) | 6070 (97.5) | 1897 (98.0) | 3632 (96.8) | 1190 (97.9) |
| Other^a^ | n (%) | 157 (2.5) | 39 (2.0) | 120 (3.2) | 26 (2.1) |
| **Birthweight (kg)** | mean (SD) | 3.3 (0.5) | 3.3 (0.5) | 3.3 (0.5) | 3.3 (0.5) |
| **Childhood social class** |  |  |  |  |  |
| Professional | n (%) | 315 (5.8) | 110 (6.3) | 111 (3.4) | 36 (3.3) |
| Intermediate | n (%) | 1356 (24.8) | 421 (24.1) | 643 (19.5) | 183 (16.8) |
| Skilled non-manual | n (%) | 696 (12.7) | 200 (11.5) | 361 (10.9) | 78 (7.1) |
| Skilled manual | n (%) | 2000 (36.6) | 649 (37.2) | 1437 (43.6) | 493 (45.2) |
| Partly skilled manual | n (%) | 737 (13.5) | 271 (15.5) | 514 (15.6) | 208 (19.1) |
| Unskilled manual | n (%) | 358 (6.6) | 95 (5.4) | 234 (7.1) | 94 (8.6) |
| **Mother left education at mandatory leaving age** |  |  |  |  |  |
| *Yes* | n (%) | 3862 (65.7) | 1233 (66.8) | 2643 (73.6) | 930 (79.0) |
| **Father left education at mandatory leaving age** |  |  |  |  |  |
| *Yes* | n (%) | 3622 (65.4) | 1161 (67.3) | 2384 (72.8) | 834 (78.4) |

^a^Other ethnicities: White other, Mixed race, Indian, Pakistani, Bangladeshi, Other Asian, Caribbean, African, Other Black, Chinese

**Table S10. Proportion of each cohort assigned to each class and odds ratios for class membership (5 classes): males**

| **BMI trajectory class membership** | *Class 1: Lowest* | | *Class 2: Middle* | | *Class 3: Increasing* | | *Class 4: Higher* | | *Class 5: Highest* | |
| --- | --- | --- | --- | --- | --- | --- | --- | --- | --- | --- |
|  | N (%) | OR  (95% CI) | N (%) | OR  (95% CI) | N (%) | OR  (95% CI) | N (%) | OR  (95% CI) | N (%) | OR  (95% CI) |
| Males (n=12465) |  | |  |  |  | |  | |  | |
| *NSHD* | 1058 (56.5) | (ref) | 370 (19.8) | (ref) | 325 (17.4) | (ref) | 109 (5.8) | (ref) | 10 (0.5) | (ref) |
| *NCDS* | 3308 (52.6) | - | 1288 (20.5) | 1.19 (0.90, 1.57) | 1110 (17.6) | 1.10 (0.83, 1.44) | 468 (7.4) | 1.37 (1.02, 1.85) | 117 (1.9) | 7.10 (1.66, 30.34) |
| *BCS* | 1971 (45.8) | - | 715 (16.6) | 0.93 (0.67, 1.29) | 1176 (27.3) | 2.86 (2.18, 3.75) | 328 (7.6) | 1.61 (1.17, 2.23) | 112 (2.6) | 12.85 (3.00, 54.98) |

**Table S11. Proportion of each cohort assigned to each class and odds ratios for class membership (5 classes): females**

| **BMI trajectory class membership** | *Class 1: Lowest* | | *Class 2: Middle* | | *Class 3: Lowest to middle* | | *Class 4: Increasing* | | *Class 5: Highest* | |
| --- | --- | --- | --- | --- | --- | --- | --- | --- | --- | --- |
|  | N (%) | OR  (95% CI) | N (%) | OR  (95% CI) | N (%) | OR  (95% CI) | N (%) | OR  (95% CI) | N (%) | OR  (95% CI) |
| Females (n=13190) |  | |  |  |  | |  | |  | |
| *NSHD* | 731 (40.1) | (ref) | 300 (16.5) | (ref) | 455 (25.0) | (ref) | 257 (14.1) | (ref) | 78 (4.3) | (ref) |
| *NCDS* | 2266 (36.4) | - | 1120 (18.0) | 1.25 (0.97, 1.61) | 1427 (22.9) | 0.93 (0.71, 1.23) | 1078 (17.3) | 1.49 (1.16, 1.91) | 337 (5.4) | 1.41 (1.02, 1.96) |
| *BCS* | 1584 (30.8) | - | 741 (14.4) | 1.07 (0.81, 1.43) | 1318 (25.6) | 1.52 (1.14, 2.03) | 1169 (22.7) | 2.81 (2.18, 3.61) | 329 (6.4) | 2.15 (1.54, 301) |
