# Supplementary material for: Changes over time in latent patterns of childhood-to-adulthood BMI development in Great Britain: evidence from three cohorts born in 1946, 1958, and 1970
Source: BMC Med. 2021 Apr 21;19:96. doi: 10.1186/s12916-021-01969-8 (PMC8059270; doi:10.1186/s12916-021-01969-8)
Supplement: Supplementary file 2 — Additional file 2: Text S1. Mplus output file: males. Text S2. Mplus output file: females. [file 12916_2021_1969_MOESM2_ESM.pdf]

```
Mplus VERSION 8.5
MUTHEN & MUTHEN
03/04/2021   9:48 PM

INPUT INSTRUCTIONS

    TITLE:  4 cubic_males;
    DATA: FILE IS "mplusdata_males_7off_v2.txt";
    VARIABLE:
         NAMES ARE
       sid sid_v21 sid_v22 sid_v23 id sex bmi1 bmi2 bmi3 bmi4 bmi5
       wtself1 wtself2 wtself3 wtself4 wtself5
       cage1 cage2 cage3 cage4 cage5;

        USEVARIABLES ARE
        bmi1 bmi2 bmi3 bmi4 bmi5;

        IDVARIABLE IS id;

        MISSING are ALL(-9999);
        CLASSES = c(4);
        AUXILIARY (R3STEP) sid_v22 sid_v23 ;

      ANALYSIS:
        ESTIMATOR = MLR;
        TYPE IS MIXTURE;
        STARTS = 1000 200;


    MODEL:
      %OVERALL%
      i s q cubic | bmi1@-12.60 bmi2@-7.8 bmi3@0 bmi4@10.29 bmi5@18.55;

      cubic@0;
      q@0;
      s WITH q @0;
      i WITH q @0;

      bmi5 ON bmi4;
      bmi4 ON bmi3;
      bmi3 ON bmi2;
      bmi2 ON bmi1;

       %c#1%
        [i](c1p1);
        [s](c1p2);
        [q](c1p3);
        [cubic](c1p4);

        bmi1-bmi5 (c1r1-c1r5);



       %c#2%
        [i](c2p1);
        [s](c2p2);
        [q](c2p3);
        [cubic](c2p4);

        bmi1-bmi5 (c2r1-c2r5);

       bmi5 ON bmi4;
       bmi4 ON bmi3;
       bmi3 ON bmi2;
       bmi2 ON bmi1;


       %c#3%
```

```
         [i](c3p1);
         [s](c3p2);
         [q](c3p3);
         [cubic](c3p4);

         bmi1-bmi5 (c3r1-c3r5);

        bmi5 ON bmi4;
        bmi4 ON bmi3;
        bmi3 ON bmi2;
        bmi2 ON bmi1;

        %c#4%
         [i](c4p1);
         [s](c4p2);
         [q](c4p3);
         [cubic](c4p4);

         bmi1-bmi5 (c4r1-c4r5);

         bmi5 ON bmi4;
         bmi4 ON bmi3;
         bmi3 ON bmi2;
         bmi2 ON bmi1;

    MODEL CONSTRAINT:
        PLOT(class1 class2 class3 class4);
        LOOP(time,11.04,42.19,0.001);
        class1 = c1p1 + c1p2*(time-23.64) + c1p3*(time-23.64)^2 + c1p4*(time-23.64)^3;
        class2 = c2p1 + c2p2*(time-23.64) + c2p3*(time-23.64)^2 + c2p4*(time-23.64)^3;
        class3 = c3p1 + c3p2*(time-23.64) + c3p3*(time-23.64)^2 + c3p4*(time-23.64)^3;
        class4 = c4p1 + c4p2*(time-23.64) + c4p3*(time-23.64)^2 + c4p4*(time-23.64)^3;


     PLOT:
        TYPE = PLOT1;
        TYPE = PLOT2;
        TYPE = PLOT3;
        SERIES = bmi1(11.04) bmi2(15.84) bmi3(23.64) bmi4(33.93) bmi5(42.19);



    SAVEDATA:
      FILE IS "four_males_auto_no_integr.dat";
      SAVE = CPROB;

    OUTPUT:
      TECH4 sampstat cinterval;



*** WARNING in MODEL command
  All continuous latent variable covariances involving Q have been fixed to 0
  because the variance of Q is fixed at 0.
*** WARNING in MODEL command
  All continuous latent variable covariances involving CUBIC have been fixed to 0
  because the variance of CUBIC is fixed at 0.
   2 WARNING(S) FOUND IN THE INPUT INSTRUCTIONS



4 cubic_males;

SUMMARY OF ANALYSIS

Number of groups                                                 1
Number of observations                                       12465
```

```
Number of dependent variables                                    5
Number of independent variables                                  0
Number of continuous latent variables                            4
Number of categorical latent variables                           1

Observed dependent variables

  Continuous
   BMI1        BMI2        BMI3        BMI4        BMI5

Observed auxiliary variables
   SID_V22     SID_V23

Continuous latent variables
   I           S           Q           CUBIC

Categorical latent variables
   C

Variables with special functions

  ID variable           ID

Estimator                                                      MLR
Information matrix                                        OBSERVED
Optimization Specifications for the Quasi-Newton Algorithm for
Continuous Outcomes
  Maximum number of iterations                                 100
  Convergence criterion                                  0.100D-05
Optimization Specifications for the EM Algorithm
  Maximum number of iterations                                 500
  Convergence criteria
    Loglikelihood change                                 0.100D-06
    Relative loglikelihood change                        0.100D-06
    Derivative                                           0.100D-05
Optimization Specifications for the M step of the EM Algorithm for
Categorical Latent variables
  Number of M step iterations                                    1
  M step convergence criterion                           0.100D-05
  Basis for M step termination                           ITERATION
Optimization Specifications for the M step of the EM Algorithm for
Censored, Binary or Ordered Categorical (Ordinal), Unordered
Categorical (Nominal) and Count Outcomes
  Number of M step iterations                                    1
  M step convergence criterion                           0.100D-05
  Basis for M step termination                           ITERATION
  Maximum value for logit thresholds                            15
  Minimum value for logit thresholds                           -15
  Minimum expected cell size for chi-square              0.100D-01
Maximum number of iterations for H1                           2000
Convergence criterion for H1                             0.100D-03
Optimization algorithm                                         EMA
Random Starts Specifications
  Number of initial stage random starts                       1000
  Number of final stage optimizations                          200
  Number of initial stage iterations                            10
  Initial stage convergence criterion                    0.100D+01
  Random starts scale                                    0.500D+01
  Random seed for generating random starts                       0

Input data file(s)
  mplusdata_males_7off_v2.txt
Input data format  FREE


SUMMARY OF DATA

     Number of missing data patterns            16
```

Number of y missing data patterns 16
Number of u missing data patterns 0

COVARIANCE COVERAGE OF DATA

Minimum covariance coverage value 0.100

PROPORTION OF DATA PRESENT FOR Y

Covariance Coverage

	BMI1	BMI2	BMI3	BMI4	BMI5
BMI1	0.859				
BMI2	0.647	0.744			
BMI3	0.630	0.560	0.749		
BMI4	0.700	0.594	0.611	0.830	
BMI5	0.687	0.584	0.597	0.711	0.816

SAMPLE STATISTICS

ESTIMATED SAMPLE STATISTICS

Means

BMI1	BMI2	BMI3	BMI4	BMI5
17.108	20.347	23.511	25.771	26.702

Covariances

	BMI1	BMI2	BMI3	BMI4	BMI5
BMI1	5.000				
BMI2	4.153	7.589			
BMI3	3.243	5.253	9.434		
BMI4	3.946	6.041	9.093	15.066	
BMI5	3.816	5.918	9.227	13.660	17.262

Correlations

	BMI1	BMI2	BMI3	BMI4	BMI5
BMI1	1.000				
BMI2	0.674	1.000			
BMI3	0.472	0.621	1.000		
BMI4	0.455	0.565	0.763	1.000	
BMI5	0.411	0.517	0.723	0.847	1.000

MAXIMUM LOG-LIKELIHOOD VALUE FOR THE UNRESTRICTED (H1) MODEL IS -113257.178

UNIVARIATE SAMPLE STATISTICS

UNIVARIATE HIGHER-ORDER MOMENT DESCRIPTIVE STATISTICS

Variable/ Sample Size	Mean/ Variance	Skewness/ Kurtosis	Minimum/ Maximum	% with Min/Max	Percentiles 20%/60%	40%/80%	Median
BMI1	17.098	1.555	10.273	0.01%	15.444	16.28	

```
9     16.690
          10707.000       4.946       4.451      32.881    0.01%      17.143     18.41
0
     BMI2                20.335       1.402      10.759    0.01%      18.215     19.39
4     19.935
           9279.000       7.608       4.487      43.948    0.01%      20.523     22.12
8
     BMI3                23.434       1.173      13.812    0.01%      21.092     22.36
4     23.093
           9341.000       9.194       3.621      48.976    0.01%      23.738     25.54
4
     BMI4                25.763       1.012      11.576    0.01%      22.742     24.46
2     25.256
          10350.000      14.801       2.560      50.219    0.01%      26.126     28.43
3
     BMI5                26.673       1.146      13.918    0.01%      23.424     25.25
2     26.114
          10174.000      16.995       3.026      56.491    0.01%      27.050     29.48
1
```

RANDOM STARTS RESULTS RANKED FROM THE BEST TO THE WORST LOGLIKELIHOOD VALUES

1 perturbed starting value run(s) did not converge in the initial stage
optimizations.

Final stage loglikelihood values at local maxima, seeds, and initial stage start number
s:

```
         -108600.343  109946           771
         -108600.343  637095           207
         -108600.343  534193           689
         -108600.343  608460           244
         -108600.343  621542           375
         -108600.343  420910           928
         -108600.343  980970           894
         -108600.343  535303           923
         -108600.343  150531           154
         -108600.343  79212            517
         -108600.343  987560           985
         -108600.343  153053           378
         -108600.343  407168           44
         -108600.343  395754           388
         -108600.343  94573            983
         -108600.343  512403           719
         -108600.343  345974           622
         -108600.343  76451            211
         -108600.343  92091            649
         -108600.343  91231            727
         -108600.343  847088           750
         -108600.343  43523            297
         -108600.343  843555           952
         -108600.343  425929           508
         -108600.343  954354           840
         -108600.343  966603           919
         -108600.343  486646           586
         -108600.343  650371           14
         -108600.343  548673           732
         -108600.343  237332           661
         -108656.386  484116           915
         -108656.386  970689           266
         -113404.345  945065           255
         -113404.345  76974            16
         -113404.345  496881           192
         -113404.345  710154           831
         -113404.345  322790           636
         -113404.345  982520           737
         -113404.345  717754           180
         -113404.345  830529           279
```

```
          -113404.345  155622           507
          -113404.345  78862            529
          -113404.345  701525           239
          -113404.345  800454           751
          -113404.345  814975           129
          -113404.345  520177           262
          -113404.345  93468            3
          -113404.345  922596           456
          -113404.345  92689            304
          -113404.345  107446           12
          -113404.345  546943           865
          -113404.345  700846           867
          -113404.345  276102           599
          -113404.345  723775           97
          -113404.345  674171           195
          -113404.345  350608           334
          -113404.345  391368           802
          -113404.345  208620           965
          -113404.345  358488           264
          -113404.345  488125           368
          -113404.345  73576            213
          -113404.345  677062           680
          -113404.345  791678           974
          -113404.345  55115            408
          -113404.345  68850            462
          -113404.345  695155           150
          -113404.345  193569           440
          -113404.345  802682           419
          -113404.345  387701           275
          -113404.345  928287           197
          -113404.345  499150           216
          -113404.345  939709           112
          -113404.345  689529           516
          -113404.345  939870           655
          -113404.345  462821           745
          -113404.345  105656           909
          -113404.345  860772           174
          -113404.345  900921           984
          -113404.345  781489           627
          -113404.345  727176           861
          -113404.345  213532           503
          -113404.345  127362           757
          -113404.345  910224           580
          -113404.345  645664           39
          -113404.345  298553           773
          -113404.345  972430           491
          -113404.345  567165           319
          -113404.345  626087           314
          -113404.345  580181           691
          -113404.345  260601           36
          -113404.345  572637           989
          -113404.345  618760           489
          -113404.345  831410           567
          -113404.345  821011           161
          -113404.345  97300            640
          -113404.345  232226           235
          -113404.345  816765           959
          -113404.345  248742           556
          -113404.345  607072           717
          -113404.345  265218           924
          -113404.345  810705           626
          -113404.345  608849           224
          -113404.345  130541           896
          -113404.345  376411           473
          -113404.345  165853           105
          -113404.345  370466           41
          -113404.345  317868           740
          -113488.134  772131           407
```

```
         -113488.134  167409           772

91 perturbed starting value run(s) did not converge or were rejected in the third stage
.



THE BEST LOGLIKELIHOOD VALUE HAS BEEN REPLICATED.  RERUN WITH AT LEAST TWICE THE
RANDOM STARTS TO CHECK THAT THE BEST LOGLIKELIHOOD IS STILL OBTAINED AND REPLICATED.


THE MODEL ESTIMATION TERMINATED NORMALLY



MODEL FIT INFORMATION

Number of Free Parameters                       58

Loglikelihood

          H0 Value                        -108600.343
          H0 Scaling Correction Factor      1.2133
            for MLR

Information Criteria

          Akaike (AIC)                     217316.686
          Bayesian (BIC)                   217747.666
          Sample-Size Adjusted BIC         217563.348
            (n* = (n + 2) / 24)



FINAL CLASS COUNTS AND PROPORTIONS FOR THE LATENT CLASSES
BASED ON THE ESTIMATED MODEL

    Latent
   Classes

       1        905.41341          0.07264
       2       3003.59832          0.24096
       3       2614.82674          0.20977
       4       5941.16153          0.47663


FINAL CLASS COUNTS AND PROPORTIONS FOR THE LATENT CLASSES
BASED ON ESTIMATED POSTERIOR PROBABILITIES

    Latent
   Classes

       1        905.41341          0.07264
       2       3003.59832          0.24096
       3       2614.82674          0.20977
       4       5941.16153          0.47663


FINAL CLASS COUNTS AND PROPORTIONS FOR THE LATENT CLASSES
BASED ON THEIR MOST LIKELY LATENT CLASS MEMBERSHIP

Class Counts and Proportions

    Latent
   Classes

       1              718          0.05760
       2             2561          0.20546
```

3	2032	0.16302
4	7154	0.57393

CLASSIFICATION QUALITY

Entropy	0.505

Average Latent Class Probabilities for Most Likely Latent Class Membership (Row)
by Latent Class (Column)

	1	2	3	4
1	0.836	0.083	0.081	0.000
2	0.061	0.679	0.128	0.133
3	0.065	0.129	0.688	0.119
4	0.003	0.132	0.116	0.749

Classification Probabilities for the Most Likely Latent Class Membership (Column)
by Latent Class (Row)

	1	2	3	4
1	0.663	0.171	0.145	0.021
2	0.020	0.579	0.087	0.314
3	0.022	0.125	0.534	0.318
4	0.000	0.057	0.041	0.902

Logits for the Classification Probabilities for the Most Likely Latent Class Membership
(Column)
by Latent Class (Row)

	1	2	3	4
1	3.469	2.116	1.952	0.000
2	-2.757	0.612	-1.282	0.000
3	-2.663	-0.936	0.518	0.000
4	-11.408	-2.756	-3.102	0.000

MODEL RESULTS

	Estimate	S.E.	Est./S.E.	Two-Tailed P-Value
**Latent Class 1**				
I |				
BMI1	1.000	0.000	999.000	999.000
BMI2	1.000	0.000	999.000	999.000
BMI3	1.000	0.000	999.000	999.000
BMI4	1.000	0.000	999.000	999.000
BMI5	1.000	0.000	999.000	999.000
S |				
BMI1	-12.600	0.000	999.000	999.000
BMI2	-7.800	0.000	999.000	999.000
BMI3	0.000	0.000	999.000	999.000
BMI4	10.290	0.000	999.000	999.000
BMI5	18.550	0.000	999.000	999.000
Q |				
BMI1	158.760	0.000	999.000	999.000
BMI2	60.840	0.000	999.000	999.000
BMI3	0.000	0.000	999.000	999.000

```
    BMI4             105.884      0.000    999.000    999.000
    BMI5             344.102      0.000    999.000    999.000

 CUBIC    |
    BMI1           -2000.376      0.000    999.000    999.000
    BMI2            -474.552      0.000    999.000    999.000
    BMI3               0.000      0.000    999.000    999.000
    BMI4            1089.547      0.000    999.000    999.000
    BMI5            6383.101      0.000    999.000    999.000

 BMI5       ON
    BMI4               0.618      0.040     15.340      0.000

 BMI4       ON
    BMI3               0.581      0.058      9.991      0.000

 BMI3       ON
    BMI2               0.437      0.041     10.757      0.000

 BMI2       ON
    BMI1               0.324      0.056      5.799      0.000

 S        WITH
    Q                  0.000      0.000    999.000    999.000
    I                  0.058      0.009      6.717      0.000

 I        WITH
    Q                  0.000      0.000    999.000    999.000

 Means
    I                 16.914      1.025     16.506      0.000
    S                 -0.163      0.207     -0.788      0.431
    Q                  0.006      0.008      0.733      0.464
    CUBIC              0.000      0.001     -0.438      0.662

 Intercepts
    BMI1               0.000      0.000    999.000    999.000
    BMI2               0.000      0.000    999.000    999.000
    BMI3               0.000      0.000    999.000    999.000
    BMI4               0.000      0.000    999.000    999.000
    BMI5               0.000      0.000    999.000    999.000

 Variances
    I                  1.760      0.132     13.367      0.000
    S                  0.005      0.001      8.259      0.000
    Q                  0.000      0.000    999.000    999.000
    CUBIC              0.000      0.000    999.000    999.000

 Residual Variances
    BMI1              12.009      0.864     13.893      0.000
    BMI2              14.756      1.430     10.322      0.000
    BMI3              15.172      1.810      8.383      0.000
    BMI4              19.805      2.470      8.020      0.000
    BMI5              12.570      1.646      7.637      0.000

Latent Class 2

 I        |
    BMI1               1.000      0.000    999.000    999.000
    BMI2               1.000      0.000    999.000    999.000
    BMI3               1.000      0.000    999.000    999.000
    BMI4               1.000      0.000    999.000    999.000
    BMI5               1.000      0.000    999.000    999.000

 S        |
    BMI1             -12.600      0.000    999.000    999.000
    BMI2              -7.800      0.000    999.000    999.000
    BMI3               0.000      0.000    999.000    999.000
```

```
   BMI4              10.290      0.000    999.000    999.000
   BMI5              18.550      0.000    999.000    999.000

 Q        |
   BMI1             158.760      0.000    999.000    999.000
   BMI2              60.840      0.000    999.000    999.000
   BMI3               0.000      0.000    999.000    999.000
   BMI4             105.884      0.000    999.000    999.000
   BMI5             344.102      0.000    999.000    999.000

 CUBIC    |
   BMI1           -2000.376      0.000    999.000    999.000
   BMI2            -474.552      0.000    999.000    999.000
   BMI3               0.000      0.000    999.000    999.000
   BMI4            1089.547      0.000    999.000    999.000
   BMI5            6383.101      0.000    999.000    999.000

 BMI5       ON
   BMI4               0.285      0.050      5.708      0.000

 BMI4       ON
   BMI3               0.322      0.041      7.898      0.000

 BMI3       ON
   BMI2               0.468      0.038     12.368      0.000

 BMI2       ON
   BMI1               0.328      0.037      8.774      0.000

 S        WITH
   Q                  0.000      0.000    999.000    999.000
   I                  0.058      0.009      6.717      0.000

 I        WITH
   Q                  0.000      0.000    999.000    999.000

 Means
   I                 15.395      0.749     20.565      0.000
   S                  0.314      0.092      3.398      0.001
   Q                  0.020      0.005      4.308      0.000
   CUBIC             -0.001      0.000     -2.826      0.005

 Intercepts
   BMI1               0.000      0.000    999.000    999.000
   BMI2               0.000      0.000    999.000    999.000
   BMI3               0.000      0.000    999.000    999.000
   BMI4               0.000      0.000    999.000    999.000
   BMI5               0.000      0.000    999.000    999.000

 Variances
   I                  1.760      0.132     13.367      0.000
   S                  0.005      0.001      8.259      0.000
   Q                  0.000      0.000    999.000    999.000
   CUBIC              0.000      0.000    999.000    999.000

 Residual Variances
   BMI1               1.165      0.241      4.826      0.000
   BMI2               2.241      0.291      7.695      0.000
   BMI3               5.141      0.296     17.369      0.000
   BMI4               5.952      0.658      9.041      0.000
   BMI5               4.099      0.489      8.389      0.000

Latent Class 3

 I        |
   BMI1               1.000      0.000    999.000    999.000
   BMI2               1.000      0.000    999.000    999.000
   BMI3               1.000      0.000    999.000    999.000
```

```
   BMI4               1.000      0.000    999.000    999.000
   BMI5               1.000      0.000    999.000    999.000

 S        |
   BMI1             -12.600      0.000    999.000    999.000
   BMI2              -7.800      0.000    999.000    999.000
   BMI3               0.000      0.000    999.000    999.000
   BMI4              10.290      0.000    999.000    999.000
   BMI5              18.550      0.000    999.000    999.000

 Q        |
   BMI1             158.760      0.000    999.000    999.000
   BMI2              60.840      0.000    999.000    999.000
   BMI3               0.000      0.000    999.000    999.000
   BMI4             105.884      0.000    999.000    999.000
   BMI5             344.102      0.000    999.000    999.000

 CUBIC    |
   BMI1           -2000.376      0.000    999.000    999.000
   BMI2            -474.552      0.000    999.000    999.000
   BMI3               0.000      0.000    999.000    999.000
   BMI4            1089.547      0.000    999.000    999.000
   BMI5            6383.101      0.000    999.000    999.000

 BMI5       ON
   BMI4               0.268      0.065      4.128      0.000

 BMI4       ON
   BMI3               0.289      0.060      4.834      0.000

 BMI3       ON
   BMI2               0.212      0.035      6.112      0.000

 BMI2       ON
   BMI1               0.158      0.030      5.286      0.000

 S        WITH
   Q                  0.000      0.000    999.000    999.000
   I                  0.058      0.009      6.717      0.000

 I        WITH
   Q                  0.000      0.000    999.000    999.000

 Means
   I                 19.000      0.691     27.490      0.000
   S                  0.019      0.106      0.179      0.858
   Q                 -0.001      0.004     -0.248      0.804
   CUBIC              0.000      0.000      0.346      0.729

 Intercepts
   BMI1               0.000      0.000    999.000    999.000
   BMI2               0.000      0.000    999.000    999.000
   BMI3               0.000      0.000    999.000    999.000
   BMI4               0.000      0.000    999.000    999.000
   BMI5               0.000      0.000    999.000    999.000

 Variances
   I                  1.760      0.132     13.367      0.000
   S                  0.005      0.001      8.259      0.000
   Q                  0.000      0.000    999.000    999.000
   CUBIC              0.000      0.000    999.000    999.000

 Residual Variances
   BMI1               4.344      0.495      8.774      0.000
   BMI2               3.361      0.359      9.357      0.000
   BMI3               2.012      0.277      7.255      0.000
   BMI4               2.490      0.394      6.325      0.000
   BMI5               1.066      0.222      4.812      0.000
```

Latent Class 4

```
 I        |
    BMI1               1.000      0.000    999.000    999.000
    BMI2               1.000      0.000    999.000    999.000
    BMI3               1.000      0.000    999.000    999.000
    BMI4               1.000      0.000    999.000    999.000
    BMI5               1.000      0.000    999.000    999.000

 S        |
    BMI1             -12.600      0.000    999.000    999.000
    BMI2              -7.800      0.000    999.000    999.000
    BMI3               0.000      0.000    999.000    999.000
    BMI4              10.290      0.000    999.000    999.000
    BMI5              18.550      0.000    999.000    999.000

 Q        |
    BMI1             158.760      0.000    999.000    999.000
    BMI2              60.840      0.000    999.000    999.000
    BMI3               0.000      0.000    999.000    999.000
    BMI4             105.884      0.000    999.000    999.000
    BMI5             344.102      0.000    999.000    999.000

 CUBIC    |
    BMI1           -2000.376      0.000    999.000    999.000
    BMI2            -474.552      0.000    999.000    999.000
    BMI3               0.000      0.000    999.000    999.000
    BMI4            1089.547      0.000    999.000    999.000
    BMI5            6383.101      0.000    999.000    999.000

 BMI5       ON
    BMI4               0.027      0.070      0.377      0.706

 BMI4       ON
    BMI3               0.203      0.046      4.423      0.000

 BMI3       ON
    BMI2               0.183      0.027      6.876      0.000

 BMI2       ON
    BMI1               0.071      0.020      3.469      0.001

 S        WITH
    Q                  0.000      0.000    999.000    999.000
    I                  0.058      0.009      6.717      0.000

 I        WITH
    Q                  0.000      0.000    999.000    999.000

 Means
    I                 18.596      0.505     36.808      0.000
    S                 -0.002      0.058     -0.028      0.978
    Q                 -0.003      0.002     -1.115      0.265
    CUBIC              0.001      0.000      5.502      0.000

 Intercepts
    BMI1               0.000      0.000    999.000    999.000
    BMI2               0.000      0.000    999.000    999.000
    BMI3               0.000      0.000    999.000    999.000
    BMI4               0.000      0.000    999.000    999.000
    BMI5               0.000      0.000    999.000    999.000

 Variances
    I                  1.760      0.132     13.367      0.000
    S                  0.005      0.001      8.259      0.000
    Q                  0.000      0.000    999.000    999.000
    CUBIC              0.000      0.000    999.000    999.000
```

```
 Residual Variances
    BMI1               0.381      0.058      6.604      0.000
    BMI2               1.258      0.063     20.097      0.000
    BMI3               1.704      0.120     14.152      0.000
    BMI4               1.161      0.170      6.831      0.000
    BMI5               0.826      0.105      7.878      0.000

Categorical Latent Variables

 Means
    C#1               -1.881      0.186    -10.102      0.000
    C#2               -0.682      0.131     -5.197      0.000
    C#3               -0.821      0.153     -5.373      0.000


QUALITY OF NUMERICAL RESULTS

     Condition Number for the Information Matrix              0.608E-08
       (ratio of smallest to largest eigenvalue)


TESTS OF CATEGORICAL LATENT VARIABLE MULTINOMIAL LOGISTIC REGRESSIONS USING
THE 3-STEP PROCEDURE

                                                           Two-Tailed
                    Estimate       S.E.  Est./S.E.    P-Value

 C#1      ON
    SID_V22            0.801      0.192      4.165      0.000
    SID_V23            1.300      0.196      6.646      0.000

 C#2      ON
    SID_V22           -0.011      0.133     -0.082      0.935
    SID_V23            1.024      0.131      7.794      0.000

 C#3      ON
    SID_V22            0.197      0.120      1.644      0.100
    SID_V23            0.147      0.138      1.065      0.287

 Intercepts
    C#1               -2.761      0.181    -15.283      0.000
    C#2               -1.079      0.115     -9.401      0.000
    C#3               -0.971      0.106     -9.182      0.000

Parameterization using Reference Class 1

 C#2      ON
    SID_V22           -0.812      0.235     -3.454      0.001
    SID_V23           -0.277      0.233     -1.190      0.234

 C#3      ON
    SID_V22           -0.604      0.227     -2.655      0.008
    SID_V23           -1.154      0.238     -4.857      0.000

 C#4      ON
    SID_V22           -0.801      0.192     -4.165      0.000
    SID_V23           -1.300      0.196     -6.646      0.000

 Intercepts
    C#2                1.681      0.216      7.784      0.000
    C#3                1.789      0.212      8.458      0.000
    C#4                2.761      0.181     15.283      0.000

Parameterization using Reference Class 2

 C#1      ON
    SID_V22            0.812      0.235      3.454      0.001
```

```
   SID_V23             0.277      0.233      1.190      0.234

 C#3      ON
   SID_V22             0.208      0.169      1.232      0.218
   SID_V23            -0.877      0.174     -5.052      0.000

 C#4      ON
   SID_V22             0.011      0.133      0.082      0.935
   SID_V23            -1.024      0.131     -7.794      0.000

 Intercepts
   C#1                -1.681      0.216     -7.784      0.000
   C#3                 0.108      0.147      0.733      0.464
   C#4                 1.079      0.115      9.401      0.000

Parameterization using Reference Class 3

 C#1      ON
   SID_V22             0.604      0.227      2.655      0.008
   SID_V23             1.154      0.238      4.857      0.000

 C#2      ON
   SID_V22            -0.208      0.169     -1.232      0.218
   SID_V23             0.877      0.174      5.052      0.000

 C#4      ON
   SID_V22            -0.197      0.120     -1.644      0.100
   SID_V23            -0.147      0.138     -1.065      0.287

 Intercepts
   C#1                -1.789      0.212     -8.458      0.000
   C#2                -0.108      0.147     -0.733      0.464
   C#4                 0.971      0.106      9.182      0.000


ODDS RATIOS FOR TESTS OF CATEGORICAL LATENT VARIABLE MULTINOMIAL LOGISTIC REGRESSIONS
USING THE 3-STEP PROCEDURE

                                                 95% C.I.
                    Estimate       S.E. Lower 2.5% Upper 2.5%

 C#1      ON
   SID_V22             2.227      0.428      1.528      3.246
   SID_V23             3.671      0.718      2.502      5.387

 C#2      ON
   SID_V22             0.989      0.132      0.761      1.285
   SID_V23             2.784      0.366      2.152      3.601

 C#3      ON
   SID_V22             1.218      0.146      0.963      1.540
   SID_V23             1.158      0.160      0.884      1.517


Parameterization using Reference Class 1

 C#2      ON
   SID_V22             0.444      0.104      0.280      0.704
   SID_V23             0.758      0.176      0.481      1.196

 C#3      ON
   SID_V22             0.547      0.124      0.350      0.854
   SID_V23             0.315      0.075      0.198      0.503

 C#4      ON
   SID_V22             0.449      0.086      0.308      0.655
   SID_V23             0.272      0.053      0.186      0.400
```

Parameterization using Reference Class 2

C#1 ON				
SID_V22	2.251	0.529	1.421	3.568
SID_V23	1.319	0.307	0.836	2.081
C#3 ON				
SID_V22	1.231	0.208	0.885	1.713
SID_V23	0.416	0.072	0.296	0.585
C#4 ON				
SID_V22	1.011	0.135	0.778	1.313
SID_V23	0.359	0.047	0.278	0.465

Parameterization using Reference Class 3

C#1 ON				
SID_V22	1.829	0.416	1.171	2.856
SID_V23	3.170	0.753	1.990	5.049
C#2 ON				
SID_V22	0.812	0.137	0.584	1.131
SID_V23	2.403	0.417	1.710	3.377
C#4 ON				
SID_V22	0.821	0.098	0.649	1.039
SID_V23	0.863	0.119	0.659	1.131

CONFIDENCE INTERVALS OF MODEL RESULTS

	Lower .5%	Lower 2.5%	Lower 5%	Estimate	Upper 5%	Upper 2.5%	Upper .5%
Latent Class 1							
I |							
BMI1	1.000	1.000	1.000	1.000	1.000	1.000	1.000
BMI2	1.000	1.000	1.000	1.000	1.000	1.000	1.000
BMI3	1.000	1.000	1.000	1.000	1.000	1.000	1.000
BMI4	1.000	1.000	1.000	1.000	1.000	1.000	1.000
BMI5	1.000	1.000	1.000	1.000	1.000	1.000	1.000
S |							
BMI1	-12.600	-12.600	-12.600	-12.600	-12.600	-12.600	-12.600
BMI2	-7.800	-7.800	-7.800	-7.800	-7.800	-7.800	-7.800
BMI3	0.000	0.000	0.000	0.000	0.000	0.000	0.000
BMI4	10.290	10.290	10.290	10.290	10.290	10.290	10.290
BMI5	18.550	18.550	18.550	18.550	18.550	18.550	18.550
Q |							
BMI1	158.760	158.760	158.760	158.760	158.760	158.760	158.760
BMI2	60.840	60.840	60.840	60.840	60.840	60.840	60.840
BMI3	0.000	0.000	0.000	0.000	0.000	0.000	0.000
BMI4	105.884	105.884	105.884	105.884	105.884	105.884	

Text S2. Mplus output file: females


```
Mplus VERSION 8.5
MUTHEN & MUTHEN
03/04/2021  10:18 PM

INPUT INSTRUCTIONS

    TITLE:  4 cubic females;
    DATA: FILE IS "mplusdata_females_7off_v2.txt";
    VARIABLE:
         NAMES ARE
       sid sid_v21 sid_v22 sid_v23 id sex bmi1 bmi2 bmi3 bmi4 bmi5
       wtself1 wtself2 wtself3 wtself4 wtself5
       cage1 cage2 cage3 cage4 cage5;

        USEVARIABLES ARE
        bmi1 bmi2 bmi3 bmi4 bmi5;

        IDVARIABLE IS id;

        MISSING are ALL(-9999);
        CLASSES = c(4);
        AUXILIARY (R3STEP) sid_v22 sid_v23 ;

      ANALYSIS:
        ESTIMATOR = MLR;
        TYPE IS MIXTURE;
        STARTS = 1000 200;

    MODEL:
      %OVERALL%
      i s q cubic | bmi1@-15.08 bmi2@-10.12 bmi3@0 bmi4@7.94 bmi5@16.19;

      cubic@0;
      q@0;
      s WITH q @0;
      i WITH q @0;

      bmi5 ON bmi4;
      bmi4 ON bmi3;
      bmi3 ON bmi2;
      bmi2 ON bmi1;

       %c#1%
        [i](c1p1);
        [s](c1p2);
        [q](c1p3);
        [cubic](c1p4);

        bmi1-bmi5 (c1r1-c1r5);



       %c#2%
        [i](c2p1);
        [s](c2p2);
        [q](c2p3);
        [cubic](c2p4);

        bmi1-bmi5 (c2r1-c2r5);

       bmi5 ON bmi4;
       bmi4 ON bmi3;
       bmi3 ON bmi2;
       bmi2 ON bmi1;


       %c#3%
        [i](c3p1);
```

```
         [s](c3p2);
         [q](c3p3);
         [cubic](c3p4);

         bmi1-bmi5 (c3r1-c3r5);

        bmi5 ON bmi4;
        bmi4 ON bmi3;
        bmi3 ON bmi2;
        bmi2 ON bmi1;

        %c#4%
         [i](c4p1);
         [s](c4p2);
         [q](c4p3);
         [cubic](c4p4);

         bmi1-bmi5 (c4r1-c4r5);

         bmi5 ON bmi4;
         bmi4 ON bmi3;
         bmi3 ON bmi2;
         bmi2 ON bmi1;

    MODEL CONSTRAINT:
        PLOT(class1 class2 class3 class4);
        LOOP(time,10.92,42.19,0.001);
        class1 = c1p1 + c1p2*(time-26.00) + c1p3*(time-26.00)^2 + c1p4*(time-26.00)^3;
        class2 = c2p1 + c2p2*(time-26.00) + c2p3*(time-26.00)^2 + c2p4*(time-26.00)^3;
        class3 = c3p1 + c3p2*(time-26.00) + c3p3*(time-26.00)^2 + c3p4*(time-26.00)^3;
        class4 = c4p1 + c4p2*(time-26.00) + c4p3*(time-26.00)^2 + c4p4*(time-26.00)^3;

     PLOT:
        TYPE = PLOT1;
        TYPE = PLOT2;
        TYPE = PLOT3;
        SERIES = bmi1(10.92) bmi2(15.88) bmi3(26.00) bmi4(33.94) bmi5(42.19);

    SAVEDATA:
      FILE IS "four_females_auto_no_integr.dat";
      SAVE = CPROB;

    OUTPUT:
      TECH4 sampstat cinterval;



*** WARNING in MODEL command
  All continuous latent variable covariances involving Q have been fixed to 0
  because the variance of Q is fixed at 0.
*** WARNING in MODEL command
  All continuous latent variable covariances involving CUBIC have been fixed to 0
  because the variance of CUBIC is fixed at 0.
   2 WARNING(S) FOUND IN THE INPUT INSTRUCTIONS



4 cubic females;

SUMMARY OF ANALYSIS

Number of groups                                                 1
Number of observations                                       13190

Number of dependent variables                                    5
Number of independent variables                                  0
Number of continuous latent variables                            4
Number of categorical latent variables                           1
```

```
Observed dependent variables

  Continuous
   BMI1        BMI2        BMI3        BMI4        BMI5

Observed auxiliary variables
   SID_V22     SID_V23

Continuous latent variables
   I           S           Q           CUBIC

Categorical latent variables
   C

Variables with special functions

  ID variable           ID

Estimator                                                      MLR
Information matrix                                        OBSERVED
Optimization Specifications for the Quasi-Newton Algorithm for
Continuous Outcomes
  Maximum number of iterations                                 100
  Convergence criterion                                  0.100D-05
Optimization Specifications for the EM Algorithm
  Maximum number of iterations                                 500
  Convergence criteria
    Loglikelihood change                                 0.100D-06
    Relative loglikelihood change                        0.100D-06
    Derivative                                           0.100D-05
Optimization Specifications for the M step of the EM Algorithm for
Categorical Latent variables
  Number of M step iterations                                    1
  M step convergence criterion                           0.100D-05
  Basis for M step termination                           ITERATION
Optimization Specifications for the M step of the EM Algorithm for
Censored, Binary or Ordered Categorical (Ordinal), Unordered
Categorical (Nominal) and Count Outcomes
  Number of M step iterations                                    1
  M step convergence criterion                           0.100D-05
  Basis for M step termination                           ITERATION
  Maximum value for logit thresholds                            15
  Minimum value for logit thresholds                           -15
  Minimum expected cell size for chi-square              0.100D-01
Maximum number of iterations for H1                           2000
Convergence criterion for H1                             0.100D-03
Optimization algorithm                                         EMA
Random Starts Specifications
  Number of initial stage random starts                       1000
  Number of final stage optimizations                          200
  Number of initial stage iterations                            10
  Initial stage convergence criterion                    0.100D+01
  Random starts scale                                    0.500D+01
  Random seed for generating random starts                       0

Input data file(s)
  mplusdata_females_7off_v2.txt
Input data format  FREE


SUMMARY OF DATA

     Number of missing data patterns           16
     Number of y missing data patterns         16
     Number of u missing data patterns          0
```

COVARIANCE COVERAGE OF DATA

Minimum covariance coverage value 0.100

PROPORTION OF DATA PRESENT FOR Y

Covariance Coverage

	BMI1	BMI2	BMI3	BMI4	BMI5
BMI1	0.853				
BMI2	0.651	0.749			
BMI3	0.728	0.646	0.864		
BMI4	0.689	0.599	0.709	0.825	
BMI5	0.675	0.586	0.691	0.700	0.809

SAMPLE STATISTICS

ESTIMATED SAMPLE STATISTICS

Means

BMI1	BMI2	BMI3	BMI4	BMI5
17.369	21.083	22.593	24.599	25.695

Covariances

	BMI1	BMI2	BMI3	BMI4	BMI5
BMI1	6.457				
BMI2	5.224	9.247			
BMI3	4.463	6.952	12.660		
BMI4	5.785	8.609	13.551	23.743	
BMI5	5.472	8.298	13.885	21.788	27.559

Correlations

	BMI1	BMI2	BMI3	BMI4	BMI5
BMI1	1.000				
BMI2	0.676	1.000			
BMI3	0.494	0.643	1.000		
BMI4	0.467	0.581	0.782	1.000	
BMI5	0.410	0.520	0.743	0.852	1.000

MAXIMUM LOG-LIKELIHOOD VALUE FOR THE UNRESTRICTED (H1) MODEL IS -130804.853

UNIVARIATE SAMPLE STATISTICS

UNIVARIATE HIGHER-ORDER MOMENT DESCRIPTIVE STATISTICS

```
         Variable/         Mean/     Skewness/   Minimum/ % with                Percent
iles
        Sample Size      Variance    Kurtosis    Maximum  Min/Max      20%/60%    40%/8
0%    Median

     BMI1                17.367       1.271      10.195    0.01%      15.348     16.38
9     16.884
           11248.000       6.436       2.775      37.749    0.01%      17.449     19.09
5
     BMI2                21.067       1.355       9.881    0.01%      18.678     20.01
```

```
0     20.638
            9877.000       9.141       5.006      53.698    0.01%      21.326     23.11
4
     BMI3                 22.536       1.647      13.978    0.01%      19.850     21.21
1     21.873
           11399.000      12.321       4.846      52.338    0.01%      22.596     24.62
8
     BMI4                 24.610       1.547      11.173    0.01%      20.974     22.60
0     23.456
           10887.000      23.526       3.769      64.684    0.01%      24.462     27.61
8
     BMI5                 25.606       1.469      11.277    0.01%      21.628     23.41
5     24.412
           10667.000      26.811       3.265      58.195    0.01%      25.544     29.01
0

RANDOM STARTS RESULTS RANKED FROM THE BEST TO THE WORST LOGLIKELIHOOD VALUES

1 perturbed starting value run(s) did not converge in the initial stage
optimizations.

Final stage loglikelihood values at local maxima, seeds, and initial stage start number
s:

         -123744.396  490123           995
         -123744.396  318177           748
         -123744.396  unperturbed      0
         -123744.396  587946           120
         -123744.396  479273           156
         -123744.396  850112           922
         -123744.396  971693           470
         -123744.396  506886           576
         -123744.396  549244           756
         -123744.396  964570           701
         -123744.396  954354           840
         -123744.396  299977           956
         -123744.396  420910           928
         -123744.396  323588           826
         -123744.396  496710           386
         -123744.396  817298           967
         -123744.396  341041           34
         -123744.396  161421           519
         -123744.396  622290           880
         -123744.396  848331           137
         -123744.396  668003           647
         -123744.396  588699           828
         -123744.396  354208           196
         -123744.396  876943           650
         -123744.396  345974           622
         -123744.396  118421           139
         -123853.217  252346           528
         -123853.217  618000           190
         -123853.217  118958           994
         -123853.217  831410           567
         -123853.217  982520           737
         -123853.217  865906           641
         -130906.355  188498           258
         -130906.355  629320           222
         -130906.355  691234           250
         -130906.355  112586           494
         -130906.355  494209           904
         -130906.355  521575           313
         -130906.355  760531           550
         -130906.355  319144           176
         -130906.355  529455           268
         -130906.355  399508           415
         -130906.355  387701           275
         -130906.355  928624           981
```

-130906.355	370466	41
-130906.355	567165	319
-130906.355	168888	817
-130906.355	237332	661
-130906.355	556929	734
-130906.355	473343	844
-130906.355	945065	255
-130906.355	157351	579
-130906.355	910224	580
-130906.355	647617	725
-130906.355	366706	29
-130906.355	891347	504
-130906.355	254551	996
-130906.355	82200	830
-130906.355	298275	418
-130906.355	327927	908
-130906.355	941975	816
-130906.355	685268	596
-130906.355	484501	163
-130906.355	268217	83
-130906.355	163110	584
-130906.355	814975	129
-130906.355	495366	964
-130906.355	76451	211
-130906.355	798821	423
-130906.355	130541	896
-130906.355	114433	708
-130906.355	699810	571
-130906.355	203508	806
-130906.355	710154	831
-130906.355	193569	440
-130906.355	700846	867
-130906.355	350608	334
-130906.355	395754	388
-130906.355	802682	419
-130906.355	700270	855
-130906.355	89970	223
-130906.355	265218	924
-130906.355	7959	256
-130906.355	127362	757
-130906.355	94573	983
-130906.355	153942	31
-130906.355	879338	309
-130906.355	462821	745
-130906.355	780698	337
-130906.355	286735	175
-130906.355	79212	517
-130906.355	121425	511
-130906.355	972430	491
-130906.355	301717	823
-130938.060	35191	703
-130938.060	164305	128
-130938.060	303834	798
-130938.060	137305	379
-130938.060	628143	854
-130938.060	552272	654
-130938.060	147440	514
-130938.060	411615	171
-130938.060	765586	709
-130938.060	355254	796
-130938.060	105656	909
-130938.060	794236	127
-130938.060	810705	626
-130938.060	80438	246
-130938.060	211281	292
-130938.060	264081	186
-130938.060	926283	269
-130938.060	891531	206

```
         -130938.060  595759           997
         -130938.060  210870           383
         -130938.060  232226           235
         -130938.060  702492           718
         -130938.060  72662            729
         -130938.060  724087           925
         -130938.060  455410           999
         -130938.060  991329           182
         -130938.060  848163           47
         -130938.060  461687           833
         -130938.060  576783           866
         -130938.060  188640           451
         -130938.060  791285           416
         -130938.060  506113           895
         -130938.060  985387           381
         -130938.060  465160           862
         -130938.060  316165           299
         -130938.060  710445           792
         -130938.060  373505           88
         -130938.060  140442           500
         -130938.060  821011           161
         -130938.060  646573           741
         -130938.060  987560           985
         -130938.060  344422           296
         -130938.060  671390           814
         -130938.060  871438           561
         -130938.060  343926           624
         -130938.060  23012            352
         -130938.060  491970           563
```

59 perturbed starting value run(s) did not converge or were rejected in the third stage
.

THE BEST LOGLIKELIHOOD VALUE HAS BEEN REPLICATED. RERUN WITH AT LEAST TWICE THE
RANDOM STARTS TO CHECK THAT THE BEST LOGLIKELIHOOD IS STILL OBTAINED AND REPLICATED.

THE MODEL ESTIMATION TERMINATED NORMALLY

MODEL FIT INFORMATION

```
Number of Free Parameters                       58

Loglikelihood

          H0 Value                     -123744.396
          H0 Scaling Correction Factor      1.2564
            for MLR

Information Criteria

          Akaike (AIC)                  247604.791
          Bayesian (BIC)                248039.050
          Sample-Size Adjusted BIC      247854.731
            (n* = (n + 2) / 24)
```

FINAL CLASS COUNTS AND PROPORTIONS FOR THE LATENT CLASSES
BASED ON THE ESTIMATED MODEL

```
    Latent
   Classes
```

1	1427.39212	0.10822
2	2473.88445	0.18756
3	3921.13290	0.29728
4	5367.59054	0.40694

FINAL CLASS COUNTS AND PROPORTIONS FOR THE LATENT CLASSES
BASED ON ESTIMATED POSTERIOR PROBABILITIES

Latent Classes		
1	1427.39212	0.10822
2	2473.88445	0.18756
3	3921.13290	0.29728
4	5367.59054	0.40694

FINAL CLASS COUNTS AND PROPORTIONS FOR THE LATENT CLASSES
BASED ON THEIR MOST LIKELY LATENT CLASS MEMBERSHIP

Class Counts and Proportions

Latent Classes		
1	1227	0.09303
2	1942	0.14723
3	3771	0.28590
4	6250	0.47384

CLASSIFICATION QUALITY

Entropy 0.565

Average Latent Class Probabilities for Most Likely Latent Class Membership (Row)
by Latent Class (Column)

	1	2	3	4
1	0.862	0.031	0.107	0.000
2	0.043	0.695	0.156	0.106
3	0.073	0.107	0.741	0.078
4	0.002	0.109	0.110	0.779

Classification Probabilities for the Most Likely Latent Class Membership (Column)
by Latent Class (Row)

	1	2	3	4
1	0.741	0.058	0.193	0.008
2	0.015	0.546	0.164	0.275
3	0.033	0.077	0.713	0.176
4	0.000	0.038	0.055	0.907

Logits for the Classification Probabilities for the Most Likely Latent Class Membership (Column)
by Latent Class (Row)

	1	2	3	4
1	4.579	2.037	3.232	0.000
2	-2.896	0.683	-0.521	0.000
3	-1.662	-0.821	1.399	0.000

```
    4     -9.427   -3.166   -2.804    0.000


MODEL RESULTS

                                                    Two-Tailed
                    Estimate       S.E.  Est./S.E.    P-Value

Latent Class 1

 I        |
    BMI1               1.000      0.000    999.000    999.000
    BMI2               1.000      0.000    999.000    999.000
    BMI3               1.000      0.000    999.000    999.000
    BMI4               1.000      0.000    999.000    999.000
    BMI5               1.000      0.000    999.000    999.000

 S        |
    BMI1             -15.080      0.000    999.000    999.000
    BMI2             -10.120      0.000    999.000    999.000
    BMI3               0.000      0.000    999.000    999.000
    BMI4               7.940      0.000    999.000    999.000
    BMI5              16.190      0.000    999.000    999.000

 Q        |
    BMI1             227.406      0.000    999.000    999.000
    BMI2             102.414      0.000    999.000    999.000
    BMI3               0.000      0.000    999.000    999.000
    BMI4              63.044      0.000    999.000    999.000
    BMI5             262.116      0.000    999.000    999.000

 CUBIC    |
    BMI1           -3429.289      0.000    999.000    999.000
    BMI2           -1036.434      0.000    999.000    999.000
    BMI3               0.000      0.000    999.000    999.000
    BMI4             500.566      0.000    999.000    999.000
    BMI5            4243.660      0.000    999.000    999.000

 BMI5       ON
    BMI4               0.708      0.032     22.062      0.000

 BMI4       ON
    BMI3               0.503      0.053      9.581      0.000

 BMI3       ON
    BMI2               0.451      0.033     13.497      0.000

 BMI2       ON
    BMI1               0.477      0.050      9.465      0.000

 S        WITH
    Q                  0.000      0.000    999.000    999.000
    I                  0.026      0.004      6.363      0.000

 I        WITH
    Q                  0.000      0.000    999.000    999.000

 Means
    I                 17.089      0.929     18.403      0.000
    S                  0.438      0.199      2.202      0.028
    Q                 -0.002      0.004     -0.387      0.699
    CUBIC             -0.003      0.001     -3.829      0.000

 Intercepts
    BMI1               0.000      0.000    999.000    999.000
    BMI2               0.000      0.000    999.000    999.000
    BMI3               0.000      0.000    999.000    999.000
    BMI4               0.000      0.000    999.000    999.000
```

```
    BMI5               0.000      0.000    999.000    999.000

 Variances
    I                  1.327      0.081     16.405      0.000
    S                  0.003      0.000     12.769      0.000
    Q                  0.000      0.000    999.000    999.000
    CUBIC              0.000      0.000    999.000    999.000

 Residual Variances
    BMI1              10.790      0.681     15.844      0.000
    BMI2              12.762      1.479      8.628      0.000
    BMI3              19.835      1.455     13.631      0.000
    BMI4              26.087      1.895     13.766      0.000
    BMI5              20.327      1.743     11.660      0.000

Latent Class 2

 I        |
    BMI1               1.000      0.000    999.000    999.000
    BMI2               1.000      0.000    999.000    999.000
    BMI3               1.000      0.000    999.000    999.000
    BMI4               1.000      0.000    999.000    999.000
    BMI5               1.000      0.000    999.000    999.000

 S        |
    BMI1             -15.080      0.000    999.000    999.000
    BMI2             -10.120      0.000    999.000    999.000
    BMI3               0.000      0.000    999.000    999.000
    BMI4               7.940      0.000    999.000    999.000
    BMI5              16.190      0.000    999.000    999.000

 Q        |
    BMI1             227.406      0.000    999.000    999.000
    BMI2             102.414      0.000    999.000    999.000
    BMI3               0.000      0.000    999.000    999.000
    BMI4              63.044      0.000    999.000    999.000
    BMI5             262.116      0.000    999.000    999.000

 CUBIC    |
    BMI1           -3429.289      0.000    999.000    999.000
    BMI2           -1036.434      0.000    999.000    999.000
    BMI3               0.000      0.000    999.000    999.000
    BMI4             500.566      0.000    999.000    999.000
    BMI5            4243.660      0.000    999.000    999.000

 BMI5       ON
    BMI4               0.344      0.058      5.918      0.000

 BMI4       ON
    BMI3               0.374      0.048      7.754      0.000

 BMI3       ON
    BMI2               0.186      0.027      6.936      0.000

 BMI2       ON
    BMI1               0.106      0.036      2.968      0.003

 S        WITH
    Q                  0.000      0.000    999.000    999.000
    I                  0.026      0.004      6.363      0.000

 I        WITH
    Q                  0.000      0.000    999.000    999.000

 Means
    I                 17.995      0.567     31.745      0.000
    S                 -0.363      0.120     -3.029      0.002
    Q                 -0.003      0.003     -1.272      0.203
```

```
    CUBIC              0.001      0.000      2.467      0.014

 Intercepts
    BMI1               0.000      0.000    999.000    999.000
    BMI2               0.000      0.000    999.000    999.000
    BMI3               0.000      0.000    999.000    999.000
    BMI4               0.000      0.000    999.000    999.000
    BMI5               0.000      0.000    999.000    999.000

 Variances
    I                  1.327      0.081     16.405      0.000
    S                  0.003      0.000     12.769      0.000
    Q                  0.000      0.000    999.000    999.000
    CUBIC              0.000      0.000    999.000    999.000

 Residual Variances
    BMI1               5.090      0.432     11.788      0.000
    BMI2               4.471      0.379     11.788      0.000
    BMI3               1.870      0.195      9.584      0.000
    BMI4               2.480      0.424      5.852      0.000
    BMI5               1.652      0.366      4.511      0.000

Latent Class 3

 I        |
    BMI1               1.000      0.000    999.000    999.000
    BMI2               1.000      0.000    999.000    999.000
    BMI3               1.000      0.000    999.000    999.000
    BMI4               1.000      0.000    999.000    999.000
    BMI5               1.000      0.000    999.000    999.000

 S        |
    BMI1             -15.080      0.000    999.000    999.000
    BMI2             -10.120      0.000    999.000    999.000
    BMI3               0.000      0.000    999.000    999.000
    BMI4               7.940      0.000    999.000    999.000
    BMI5              16.190      0.000    999.000    999.000

 Q        |
    BMI1             227.406      0.000    999.000    999.000
    BMI2             102.414      0.000    999.000    999.000
    BMI3               0.000      0.000    999.000    999.000
    BMI4              63.044      0.000    999.000    999.000
    BMI5             262.116      0.000    999.000    999.000

 CUBIC    |
    BMI1           -3429.289      0.000    999.000    999.000
    BMI2           -1036.434      0.000    999.000    999.000
    BMI3               0.000      0.000    999.000    999.000
    BMI4             500.566      0.000    999.000    999.000
    BMI5            4243.660      0.000    999.000    999.000

 BMI5       ON
    BMI4               0.452      0.026     17.264      0.000

 BMI4       ON
    BMI3               0.438      0.041     10.581      0.000

 BMI3       ON
    BMI2               0.379      0.035     10.923      0.000

 BMI2       ON
    BMI1               0.293      0.030      9.757      0.000

 S        WITH
    Q                  0.000      0.000    999.000    999.000
    I                  0.026      0.004      6.363      0.000
```

```
 I        WITH
    Q                  0.000      0.000    999.000    999.000

 Means
    I                 15.451      0.662     23.341      0.000
    S                  0.005      0.093      0.058      0.954
    Q                  0.005      0.003      1.901      0.057
    CUBIC              0.000      0.000     -0.509      0.611

 Intercepts
    BMI1               0.000      0.000    999.000    999.000
    BMI2               0.000      0.000    999.000    999.000
    BMI3               0.000      0.000    999.000    999.000
    BMI4               0.000      0.000    999.000    999.000
    BMI5               0.000      0.000    999.000    999.000

 Variances
    I                  1.327      0.081     16.405      0.000
    S                  0.003      0.000     12.769      0.000
    Q                  0.000      0.000    999.000    999.000
    CUBIC              0.000      0.000    999.000    999.000

 Residual Variances
    BMI1               1.902      0.220      8.660      0.000
    BMI2               2.702      0.207     13.069      0.000
    BMI3               4.621      0.351     13.176      0.000
    BMI4               9.415      0.643     14.646      0.000
    BMI5               7.405      0.471     15.715      0.000

Latent Class 4

 I        |
    BMI1               1.000      0.000    999.000    999.000
    BMI2               1.000      0.000    999.000    999.000
    BMI3               1.000      0.000    999.000    999.000
    BMI4               1.000      0.000    999.000    999.000
    BMI5               1.000      0.000    999.000    999.000

 S        |
    BMI1             -15.080      0.000    999.000    999.000
    BMI2             -10.120      0.000    999.000    999.000
    BMI3               0.000      0.000    999.000    999.000
    BMI4               7.940      0.000    999.000    999.000
    BMI5              16.190      0.000    999.000    999.000

 Q        |
    BMI1             227.406      0.000    999.000    999.000
    BMI2             102.414      0.000    999.000    999.000
    BMI3               0.000      0.000    999.000    999.000
    BMI4              63.044      0.000    999.000    999.000
    BMI5             262.116      0.000    999.000    999.000

 CUBIC    |
    BMI1           -3429.289      0.000    999.000    999.000
    BMI2           -1036.434      0.000    999.000    999.000
    BMI3               0.000      0.000    999.000    999.000
    BMI4             500.566      0.000    999.000    999.000
    BMI5            4243.660      0.000    999.000    999.000

 BMI5       ON
    BMI4               0.183      0.050      3.620      0.000

 BMI4       ON
    BMI3               0.166      0.030      5.543      0.000

 BMI3       ON
    BMI2               0.119      0.019      6.218      0.000
```

```
 BMI2       ON
    BMI1               0.131      0.023      5.766      0.000

 S        WITH
    Q                  0.000      0.000    999.000    999.000
    I                  0.026      0.004      6.363      0.000

 I        WITH
    Q                  0.000      0.000    999.000    999.000

 Means
    I                 18.248      0.365     50.031      0.000
    S                  0.021      0.057      0.373      0.709
    Q                 -0.005      0.002     -2.434      0.015
    CUBIC              0.000      0.000      1.264      0.206

 Intercepts
    BMI1               0.000      0.000    999.000    999.000
    BMI2               0.000      0.000    999.000    999.000
    BMI3               0.000      0.000    999.000    999.000
    BMI4               0.000      0.000    999.000    999.000
    BMI5               0.000      0.000    999.000    999.000

 Variances
    I                  1.327      0.081     16.405      0.000
    S                  0.003      0.000     12.769      0.000
    Q                  0.000      0.000    999.000    999.000
    CUBIC              0.000      0.000    999.000    999.000

 Residual Variances
    BMI1               0.507      0.076      6.649      0.000
    BMI2               1.486      0.072     20.629      0.000
    BMI3               1.320      0.070     18.723      0.000
    BMI4               1.196      0.118     10.151      0.000
    BMI5               1.175      0.100     11.806      0.000

Categorical Latent Variables

 Means
    C#1               -1.325      0.129    -10.254      0.000
    C#2               -0.775      0.122     -6.324      0.000
    C#3               -0.314      0.084     -3.745      0.000


QUALITY OF NUMERICAL RESULTS

     Condition Number for the Information Matrix              0.317E-08
       (ratio of smallest to largest eigenvalue)


TESTS OF CATEGORICAL LATENT VARIABLE MULTINOMIAL LOGISTIC REGRESSIONS USING
THE 3-STEP PROCEDURE

                                                           Two-Tailed
                    Estimate       S.E.  Est./S.E.    P-Value

 C#1      ON
    SID_V22            0.454      0.130      3.505      0.000
    SID_V23            0.790      0.130      6.061      0.000

 C#2      ON
    SID_V22            0.230      0.123      1.867      0.062
    SID_V23           -0.071      0.136     -0.526      0.599

 C#3      ON
    SID_V22            0.139      0.101      1.369      0.171
    SID_V23            0.624      0.101      6.187      0.000
```

```
 Intercepts
    C#1               -1.857      0.117    -15.806      0.000
    C#2               -0.871      0.109     -8.014      0.000
    C#3               -0.634      0.088     -7.171      0.000

Parameterization using Reference Class 1

 C#2      ON
    SID_V22           -0.225      0.162     -1.390      0.164
    SID_V23           -0.861      0.170     -5.070      0.000

 C#3      ON
    SID_V22           -0.316      0.160     -1.974      0.048
    SID_V23           -0.166      0.158     -1.047      0.295

 C#4      ON
    SID_V22           -0.454      0.130     -3.505      0.000
    SID_V23           -0.790      0.130     -6.061      0.000

 Intercepts
    C#2                0.986      0.146      6.741      0.000
    C#3                1.223      0.144      8.482      0.000
    C#4                1.857      0.117     15.806      0.000

Parameterization using Reference Class 2

 C#1      ON
    SID_V22            0.225      0.162      1.390      0.164
    SID_V23            0.861      0.170      5.070      0.000

 C#3      ON
    SID_V22           -0.091      0.147     -0.620      0.536
    SID_V23            0.696      0.155      4.483      0.000

 C#4      ON
    SID_V22           -0.230      0.123     -1.867      0.062
    SID_V23            0.071      0.136      0.526      0.599

 Intercepts
    C#1               -0.986      0.146     -6.741      0.000
    C#3                0.237      0.130      1.820      0.069
    C#4                0.871      0.109      8.014      0.000

Parameterization using Reference Class 3

 C#1      ON
    SID_V22            0.316      0.160      1.974      0.048
    SID_V23            0.166      0.158      1.047      0.295

 C#2      ON
    SID_V22            0.091      0.147      0.620      0.536
    SID_V23           -0.696      0.155     -4.483      0.000

 C#4      ON
    SID_V22           -0.139      0.101     -1.369      0.171
    SID_V23           -0.624      0.101     -6.187      0.000

 Intercepts
    C#1               -1.223      0.144     -8.482      0.000
    C#2               -0.237      0.130     -1.820      0.069
    C#4                0.634      0.088      7.171      0.000


ODDS RATIOS FOR TESTS OF CATEGORICAL LATENT VARIABLE MULTINOMIAL LOGISTIC REGRESSIONS
USING THE 3-STEP PROCEDURE

                                                 95% C.I.
                    Estimate       S.E. Lower 2.5% Upper 2.5%
```

```
 C#1      ON
    SID_V22           1.575      0.204      1.222      2.031
    SID_V23           2.204      0.287      1.707      2.845

 C#2      ON
    SID_V22           1.258      0.155      0.989      1.601
    SID_V23           0.931      0.126      0.714      1.215

 C#3      ON
    SID_V22           1.149      0.116      0.942      1.401
    SID_V23           1.867      0.188      1.532      2.275


Parameterization using Reference Class 1

 C#2      ON
    SID_V22           0.799      0.129      0.582      1.097
    SID_V23           0.423      0.072      0.303      0.590

 C#3      ON
    SID_V22           0.729      0.117      0.533      0.998
    SID_V23           0.847      0.134      0.621      1.156

 C#4      ON
    SID_V22           0.635      0.082      0.492      0.818
    SID_V23           0.454      0.059      0.351      0.586

Parameterization using Reference Class 2

 C#1      ON
    SID_V22           1.252      0.202      0.912      1.719
    SID_V23           2.367      0.402      1.696      3.302

 C#3      ON
    SID_V22           0.913      0.134      0.685      1.218
    SID_V23           2.005      0.311      1.479      2.718

 C#4      ON
    SID_V22           0.795      0.098      0.625      1.012
    SID_V23           1.074      0.146      0.823      1.402

Parameterization using Reference Class 3

 C#1      ON
    SID_V22           1.371      0.219      1.002      1.877
    SID_V23           1.180      0.187      0.865      1.610

 C#2      ON
    SID_V22           1.095      0.161      0.821      1.461
    SID_V23           0.499      0.077      0.368      0.676

 C#4      ON
    SID_V22           0.871      0.088      0.714      1.062
    SID_V23           0.536      0.054      0.439      0.653


CONFIDENCE INTERVALS OF MODEL RESULTS

                  Lower .5%  Lower 2.5%    Lower 5%    Estimate    Upper 5%  Upper 2.5%
   Upper .5%

Latent Class 1

 I        |
    BMI1            1.000       1.000       1.000       1.000       1.000       1.000
      1.000
    BMI2            1.000       1.000       1.000       1.000       1.000       1.000
```
